# Characterization of the public transit air microbiome and resistome reveals geographical specificity

**DOI:** 10.1186/s40168-021-01044-7

**Published:** 2021-05-26

**Authors:** M. H. Y. Leung, X. Tong, K. O. Bøifot, D. Bezdan, D. J. Butler, D. C. Danko, J. Gohli, D. C. Green, M. T. Hernandez, F. J. Kelly, S. Levy, G. Mason-Buck, M. Nieto-Caballero, D. Syndercombe-Court, K. Udekwu, B. G. Young, C. E. Mason, M. Dybwad, P. K. H. Lee

**Affiliations:** 1 School of Energy and Environment, City University of Hong Kong, Hong Kong SAR, China; 2 Comprehensive Defence Division, Norwegian Defence Research Establishment FFI, Kjeller, Norway; 3 Department of Analytical, Environmental & Forensic Sciences, King’s College London, London, UK; 4 Department of Physiology and Biophysics, Weill Cornell Medicine, New York, NY USA; 5 Environmental Engineering Program, College of Engineering and Applied Science, University of Colorado, Boulder, CO USA; 6 HudsonAlpha Institute of Biotechnology, Huntsville, AL USA; 7 Department of Aquatic Sciences & Assessment, Swedish University of Agriculture, Uppsala, Sweden; 8 The HRH Prince Alwaleed Bin Talal Bin Abdulaziz Alsaud Institute for Computational Biomedicine, Weill Cornell Medicine, New York, NY USA; 9 The WorldQuant Initiative for Quantitative Prediction, Weill Cornell Medicine, New York, NY USA; 10 The Feil Family Brain and Mind Research Institute, Weill Cornell Medicine, New York, NY USA

**Keywords:** Microbiome, Metagenomics, Microbial ecology, Air microbiology, Bioinformatics, High-throughput sequencing

## Abstract

**Background:**

The public transit is a built environment with high occupant density across the globe, and identifying factors shaping public transit air microbiomes will help design strategies to minimize the transmission of pathogens. However, the majority of microbiome works dedicated to the public transit air are limited to amplicon sequencing, and our knowledge regarding the functional potentials and the repertoire of resistance genes (i.e. resistome) is limited. Furthermore, current air microbiome investigations on public transit systems are focused on single cities, and a multi-city assessment of the public transit air microbiome will allow a greater understanding of whether and how broad environmental, building, and anthropogenic factors shape the public transit air microbiome in an international scale. Therefore, in this study, the public transit air microbiomes and resistomes of six cities across three continents (Denver, Hong Kong, London, New York City, Oslo, Stockholm) were characterized.

**Results:**

City was the sole factor associated with public transit air microbiome differences, with diverse taxa identified as drivers for geography-associated functional potentials, concomitant with geographical differences in species- and strain-level inferred growth profiles. Related bacterial strains differed among cities in genes encoding resistance, transposase, and other functions. Sourcetracking estimated that human skin, soil, and wastewater were major presumptive resistome sources of public transit air, and adjacent public transit surfaces may also be considered presumptive sources. Large proportions of detected resistance genes were co-located with mobile genetic elements including plasmids. Biosynthetic gene clusters and city-unique coding sequences were found in the metagenome-assembled genomes.

**Conclusions:**

Overall, geographical specificity transcends multiple aspects of the public transit air microbiome, and future efforts on a global scale are warranted to increase our understanding of factors shaping the microbiome of this unique built environment.

**Supplementary Information:**

The online version contains supplementary material available at 10.1186/s40168-021-01044-7.

## Background

The built environment (BE) plays host to a diverse assemblage of microorganisms collectively termed the microbiome [1]. The advent of metagenomic sequencing has expanded our understanding of how different environmental, geographical, and anthropogenic factors shape the BE microbiome [2–5]. In particular, the recent application of shotgun metagenomics sequencing has further deepened our insights into the functional, adaptive, and resistance potentials of the indoor microbiome [5–7], as well as potential transmission events between BEs and occupants [8–10].

Of different urban BEs, public transit systems are among the most common infrastructures, through which more than 160 million individuals pass every day [11], exchanging microorganisms with each other, as well as with public transit surfaces and air. The high occupant density within a typical public transit environment may present a public health concern, by facilitating the transmission of microorganisms between commuters via fomites [12] or via airborne routes [13]. As urbanization and modernization take place, the number of individuals travelling on global public transit systems will surely increase for decades to come. Therefore, a greater understanding of the assembly mechanisms of the public transit microbiome and its repertoire of antibiotic resistance (AR) genes, i.e. resistome, as well as potential factors governing the relationships between the public transit environment, commuters, and microbial community, will pave the way towards minimizing the transmission of pathogens and the resistome in public transits [14, 15].

As much as the dire need for a comprehensive understanding of the public transit microbiome using shotgun metagenomics is appreciated, shotgun metagenomics sequencing has only been applied to profile public transit surfaces [16, 17]. These studies have collectively shed light into the dynamics of the public transit surface microbiomes and resistomes, and its potential relationships with commuters. However, as in other BEs [18], public transit surface communities represent only a partial illustration of the overall public transit microbiome. On the other hand, investigations of the public transit microbiome are limited to single cities using amplicon sequencing [19–23], which has limited our understanding of the public transit microbiome to taxonomic composition. As a result, there is currently no information regarding the functional potential and resistome profiles of public transit air, and no systematic and comprehensive study to compare and contrast public transit air microbiome across multiple cities. Characterization of the air microbiomes and resistomes across public transit systems is of paramount importance to ultimately identify building, environmental, and anthropogenic factors that have an impact on the public transit air microbiome and resistome, which will help experts in public health and engineering fields in implementing strategies to minimize occupants’ exposure to pathogens in public transits.

Recently, the Metagenomics and Metadesign of Subways and Urban Biomes (MetaSUB) International Consortium [24] have performed a large-scale characterization of the surface microbiomes and resistomes of different global public transit systems [25], providing an account of the biogeography of public transit surface microbiomes and resistomes. Here, we matched the large-scale surface study with a seminal, comprehensive characterization of the public transit air microbiomes of six geographically distinct locations (Denver, Hong Kong, London, New York, Oslo, and Stockholm) by using shotgun metagenomics, combined with standardized air sampling and centralized sample processing and bioinformatics methodologies. We hypothesize that geographical specificity transcends multiple aspects of the public transit air microbiome, from community composition to functional and resistome profiles.

## Results

### Overall community overview of the public transit air microbiome

**Fig. 1:**
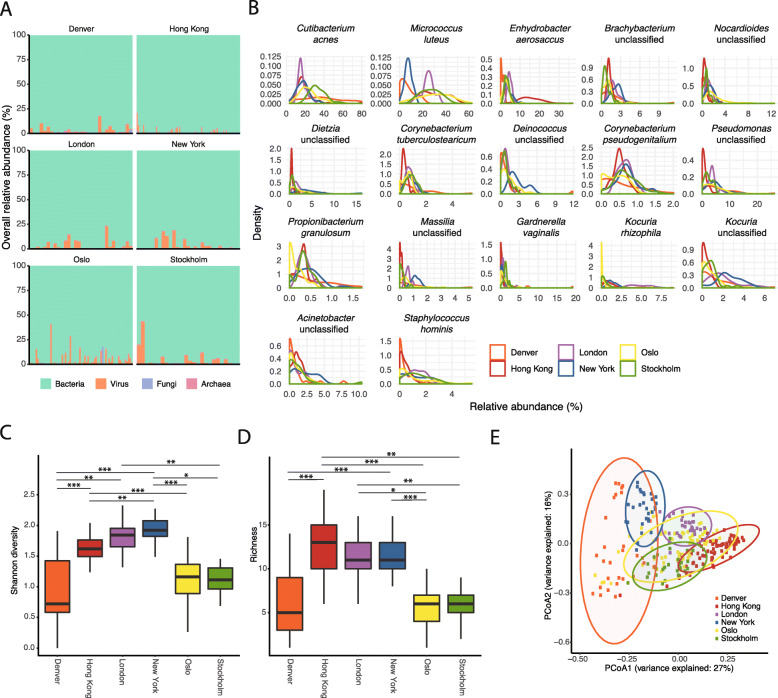
Effects of geography and related factors in driving public transit air microbiome. Colours represent each city: Denver (orange), Hong Kong (red), London (purple), New York (blue), Oslo (yellow), Stockholm (green). **a** Relative abundance of bacteria, fungi, virus, and archaea across cities. **b** Density plot of core species-level taxa (present in ≥ 75% of all samples). **c** and **d** Significant differences between **c** Shannon diversity index (Wald chi-square test *p* = 2.3 × 10^−26^) and **d** normalized richness (Wald chi-square test *p* = 5.5 × 10^−25^) of public transit air microbiomes were detected. Asterisks above horizontal bars indicate mixed model pairwise comparison significance following Tukey method *p*-value adjustment: *p < 0.05, **p < 0.01, ***p < 0.001. **e** Principal coordinates analysis plot of community composition based on Bray-Curtis dissimilarity of public transit air microbiomes grouped by city. The normal confidence ellipses indicate the confidence level at 95%

A total of 468 species-level taxa were identified in the public transit networks. As expected, the majority of the community were assigned bacteria (average relative abundance of the entire dataset (96.5%), followed by virus (3.21%), fungi (0.19%), and archaea (0.043%) (Fig. 1a). The core microbiome consisted of 17 species (species-level taxa detected in ≥ 75% of the dataset) and included commensals of human skin (*Cutibacterium acnes*, *Micrococcus luteus*, *Propionibacterium granulosum*, *Staphylococcus hominis*), as well as species of environmental origins (*Kocuria rhizophila*) (Fig. 1b). The enrichment of *Enhydrobacter aerosaccus* in Hong Kong is consistent with previous observations suggesting that the public transit air microbiome in general is influenced by the human skin [19, 20], and that members of *Enhydrobacter* may be more abundant and prevalent in Asian individuals [26, 27]. Pathogens as classified by the National Institute of Allergy and Infectious Diseases (NIAID) were not detected in this dataset.

### Public transit air microbiome exhibited geographical variations

A linear mixed model was adopted to compare microbial diversity across cities and public transit characteristics (Additional file 1: Table S1). Following normalization to 316,994 reads, both Shannon diversity and richness were significantly different among cities (Fig. 1c, d and Additional file 1: Table S1), and also between outdoor and indoor subway stations (*p* = 0.025 for Shannon, *p* = 1.0 × 10^−4^ for richness). The number of transit connections through a station was not significantly associated with Shannon diversity (*p* = 0.065) but was associated with differences in microbial richness (*p* = 0.05). Julian day and whether the public transit station was aboveground or underground were not significant predictors of microbial diversity and richness.

A nested PERMANOVA analysis revealed that city was the single greatest factor in explaining community compositional and membership differences (Additional file 1: Table S1 and Fig. 1e). A pairwise PERMANOVA analysis across the six cities also showed significant differences between all city pairs (FDR-adjusted *p* = 0.001 for all comparisons). Building design factors, ground level, indoor/outdoor, and the number of transit connections in the public transit networks had no significant influence on the overall public transit air microbiome (Additional file 1: Table S1). Julian day had also no significant effect on changes in community composition and membership. Indicator species analysis revealed that the public transit air microbiome in Hong Kong was typically characterized by high abundance of *Gordonia terrae*, and *Corynebacterium halotolerans* was uniquely enriched in the public transit air in London.

### Community- and subspecies-level in situ growth rate inference

**Fig. 2:**
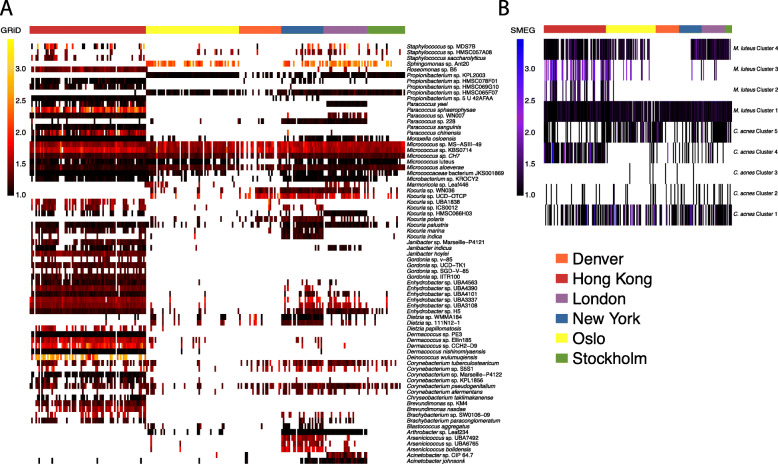
Inferred species- and strain-level growth rates showed geographically specific profiles. GRiD and SMEG were respectively applied to infer the **a** species- and **b** strain-level growth rates. GRiD was shown for species-level taxa with indices detected in greater than 10% of samples in the dataset. Samples with coverage below the default threshold for each species could not have their growth rates inferred and are indicated as white spaces on the plots

Growth Rate InDex (GRiD), a growth rate estimation method based on coverage ratios between *ori* and *ter* regions [28], was used to infer bacterial growth within the public transit air community. Overall, the majority of detected species presented low inferred growth rates (GRiD < 2.5), consistent with those inferred from BE dust [29]. Specifically, taxa of *Micrococcus* exhibited GRiD values > 1 across cities (i.e. suggestive of potential active replication), while some other genera and species were only inferred to be replicating in certain public transits (e.g. taxa of *Gordonia*, *Roseomonas*, *Dermacoccus* in Hong Kong, a number of *Kocuria*, *Dietzia*, and *Arsinicicoccus* species in New York, and *Acinetobacter* in London) (Fig. 2a). Members of *Enhydrobacter* were detected in public transits of larger metropolises (Hong Kong, London, New York), but its inferred growth appeared to be more cosmopolitan among Hong Kong metagenomes. Interestingly, taxa with the highest GRiDs are of soil and plant origins (*Sphingomonas* sp. Ant20, *Paracoccus sphaerophysae*, *Deinococcus wulumuqiensis*) (Fig. 2a).

For a given species, the GRiDs are potentially the average inferred rates of multiple strains. Therefore, to discern inferred rates of individual strains that differ by SNPs, strain-level estimation of growth (SMEG) was performed for the skin commensals *C. acnes* and *M. luteus* (Fig. 2b). SMEG showed both single strains of *M. luteus* (cluster 1) and *C. acnes* (cluster 1) being present across public transits at growth rate ratios near 1. However, metagenomes in Hong Kong had distinct strains (*M. luteus* cluster 3 and *C. acnes* cluster 4) with higher rates. These results suggest that there are potential geographical variations in bacterial growth profiles at a species level, but different closely related strains within a species may be active in a particular public transit system.

### Geographical differences in gene contents of strains associated with adaptive functions

**Fig. 3:**
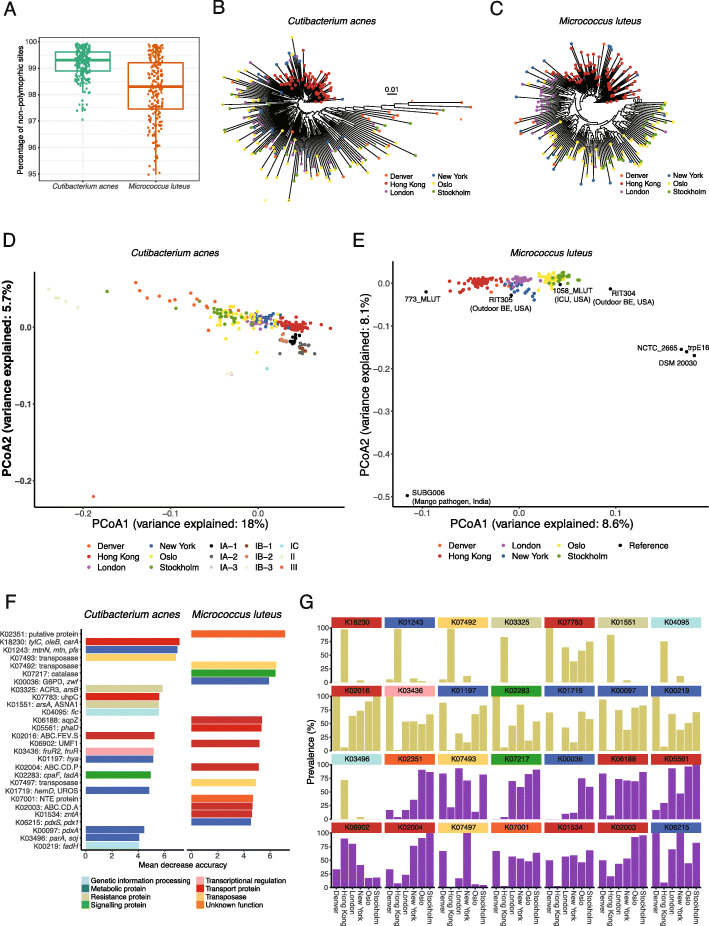
Strain-level geographical specificity in public transit air microbiome for bacteria *C. acnes* and *M. luteus* based on phylogenetic and phylogenomic analyses. **a** Percentages of non-polymorphic sites present within strains of *C. acnes* and *M. luteus* within metagenomes. **b** and **c** StrainPhlAn phylogenetic clustering of **b** *C. acnes* and **c** *M. luteus.* **d** and **e** Principal coordinates analysis plot of PanPhlAn phylogenomic gene content analysis of geography-based clustering based on Jaccard distances between strains within metagenomes. **d** *C. acnes* and **e** *M. luteus* genomes from different natural and built environments were included in the plot. **f** and **g** Geography-level KO biomarkers ranked by mean decrease in accuracy, with each KO colour coded by gene functional family (**f**), and the prevalence of the KO biomarkers in each city (light green and purple bars represent markers of *C. acnes* and *M. luteus*, respectively) (**g**)

Two skin commensals (*C. acnes* and *M. luteus*) alone made up nearly 50% of the public transit air microbiome abundance across the six cities, recapitulating results from previous studies highlighting the important influence of the skin microbiota in public transit air [19, 20]. As biogeographical patterns in human source microbiota may contribute to observed geography-based microbiome variations [18], strain-level clustering patterns of *C*. *acnes* and *M*. *luteus* were examined. StrainPhlAn phylogenetic analysis revealed extensive strain heterogeneity; strains with > 99% non-polymorphic sites (i.e. single strains) were identified in 69.5% and 29.9% of the samples in which *C. acnes* and *M. luteus* were detected, respectively (Fig. 3a). These findings suggest that multiple strains of *M. luteus* may coexist within the samples, while *C. acnes* tended to be dominated by a single strain in the majority of the samples. Geographical specificity was also inferred at the strain level for the two skin commensals (Fig. 3b, c), consistent with the release of commuter-associated microbiota, which is known to show geographical differences [18, 30].

Clustering patterns according to geography were also present at the phylogenomic level. Based on gene content, *C. acnes* strains detected in public transit air were most similar to the IA-1 and IB-2 subclades associated with individuals without acne [31] (Fig. 3d). *M. luteus* in public transit air resembled strains detected in other BEs including farms and intensive care units, and less similar to those associated with plants and type strains (Fig. 3e). Multiple metabolic and transport proteins, as well as genes encoding transposases (K07492 and K07493) and resistance (K03325 and K01551), were among the strongest markers for differentiating strains of *M. luteus* across cities (Fig. 3f, g).

### Functional potentials of public transit air microbiomes

As with community composition, city was the factor most strongly associated with functional profile differences among public transit air microbiomes (Additional file 1: Table S1). HUMAnN2 was applied to quantify the abundance of KEGG Orthologues (KOs) for the public transit air microbiomes, and revealed that 13.3% of the observed KOs (1,172/8,503) were shared by > 90% of all samples across cities. KO-based indicator feature analysis revealed that the public transit community in Hong Kong was distinctively characterized by *mtfabH* beta-ketoacyl-[acyl-carrier-protein] synthase III (K11608), mostly contributed by *G. terrae* and related members of the genus (Additional file 2: Figure S1a). Contributional diversity analysis suggested that some functional potentials may be conserved between public transit air microbiomes that are otherwise taxonomically heterogeneous (Additional file 2: Figure S1b, c and Additional file 3: Table S2).

### Identification of taxonomic drivers of functional shifts in public transit air microbiome across cities

Having identified functional differences across the public transit air microbiome, FishTaco [32] was applied to identify species estimated to drive the observed differences (Additional file 4: Figure S2 and Additional file 5: Table S3). The majority of geographical shifts were related to the metabolism of sugars, lipids, and amino acids. Interestingly, geographically-specific enrichments of functions related to the biosynthesis of secondary metabolites (geraniol (ko00281) and limonene (ko00903) in Hong Kong, carotenoid (ko00906) and stilbenoids (ko00945) in New York, and novobiocin (ko00401) in Oslo were observed. In addition, a number of pathways associated with the degradation of xenobiotic compounds nitrotoluene (ko00633) in Denver, xylene (ko00622) and caprolactam (ko00930) in Hong Kong, and bisphenol (ko00363) in New York) were detected to be among the strongest influencers of functional variations between public transits.

For each city and differential pathway, a large number of taxa appeared to drive its enrichment or attenuation, but a number of specific taxa had greater influences. Overall, the most influential driving taxa belonged to those present in all public transits but differed in abundances across cities. In Denver, enrichment and attenuation of microbial functions appeared to be driven by the skin colonizers *C. acnes* and *M. luteus* (Additional file 4: Figure S2a). In Hong Kong, the presence of the indicator species *G. terrae* drove the enrichment of a variety of pathways related to degradation of steroids, caprolactam, and limonene (Additional file 4: Figure S2b). In London, *K. rhizophila* and related species of the genus drove the enrichment of genes linked to caffeine metabolism, while *K. rhizophila* and the skin bacterium *Staphylococcus epidermidis* drove the enrichment of genes associated with d-arginine/d-ornithine metabolism (Additional file 4: Figure S2c). Also in London, enrichment of functions related to chemotaxis and flagellar assembly, as well as lipopolysaccharide biosynthesis and two-component systems, appeared to be driven by different species of *Pseudomonas*. In New York, *Pseudomonas stutzeri*, which was previously documented as the most abundant species [16], was a major influencer for the enrichment of genes related to the biosynthesis of bile acid, carotenoid, lipopolysaccharides, and polyketide sugars (Additional file 4: Figure S2d). In Oslo, enrichment of functions related to homologous recombination, pyrimidine metabolism, and pantothenate/CoA biosynthesis were contributed by *M. luteus* and a species of *Nocardioides* (Additional file 4: Figure S2e). In Stockholm, *C. acnes* appeared to drive functional shifts by the enrichment of pathways related to the biosynthesis and degradation of glycan and glycan-containing compounds, as well as simple and complex sugars (Additional file 4: Figure S2f). A full list of enriched taxa and estimated taxonomic drivers are presented in Additional file 5: Table S3.

### Public transit air resistome largely sourced from human skin, soil, and wastewater

**Fig. 4:**
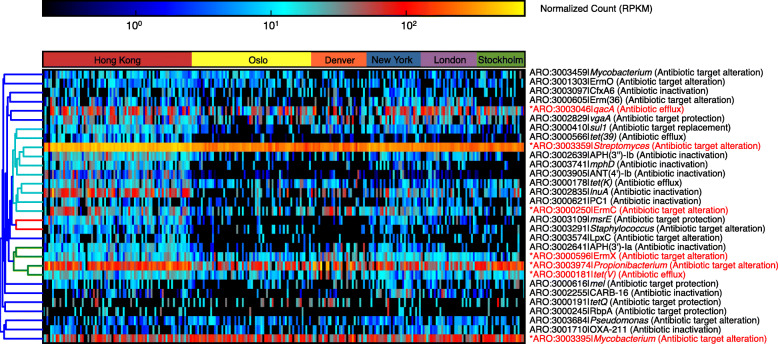
Geographical specificity in public transit air resistome. Heatmap of the top 30 AR protein families based on average reads per kilobase per million (RPKM) reads across metagenomes. Core AR protein families (those detected in ≥ 75% of the entire dataset) are indicated in red and asterisks

ShortBRED [33] identified 527 AR protein families across the public transit air microbiomes (Fig. 4). The core resistome (AR protein families detected in ≥ 75% of all samples) represented 1.3% (7/527) of the entire resistome, suggesting immense heterogeneity of resistomes across public transit networks. These core families encoded resistance against common antibiotics including aminoglycoside, elfamycin, fluoroquinolone, macrolide, and tetracycline. Consistent with our taxonomic and functional observations, geographical differences were also observed for public transit resistomes (Additional file 1: Table S1 and Additional file 6: Figure S3) (*p* = 0.02 and 0.005, *R*^2^ = 0.06 and 0.07 for Bray-Curtis dissimilarity and Jaccard distance respectively). Similar to community composition, the resistome of each city was significantly different from every other city (FDR-adjusted *p* = 0.001 for all pairwise comparisons).

To estimate the relative contributions by different potential putative resistome sources to the public transit air resistome, Bayesian sourcetracking [34] was performed by including a global collection of resistome data as putative sources. SourceTracker analysis has been used previously to infer the estimated contribution by putative sources of a resistome, based on the extent to which a user-specified resistome source overlaps with that of a metagenome sample as the sink [35]. In total, 1,402 ShortBRED markers (i.e. representative peptide sequences for protein families) were detected among the 182 source samples selected for this study. Specifically, source metagenomes from wastewater-activated sludge harboured 849 markers, which was the highest of the ecotypes included for source analysis, followed by human skin (643), animal faeces (607), human gut (495), marine sediment/water (287), human oral cavity (262), and soil (251).

Resistomes of human skin, soil, and wastewater significantly overlapped with public transit air, accounting on average for 35.5 ± 15.6%, 31.9 ± 19.1%, and 15.6 ± 12.8%, respectively (Additional file 7: Figure S4). Human oral cavity (1.1 ± 3.0%) and gut (0.8 ± 2.5%) overlapped minimally with the AR genes detected in all cities. In addition, AR genes originating from animal faeces shared a higher proportion of the air resistome in Denver than other cities, while Hong Kong and Stockholm appeared to harbour a sizeable fraction of AR genes that may have been sourced from aquatic environments.

**Fig. 5:**
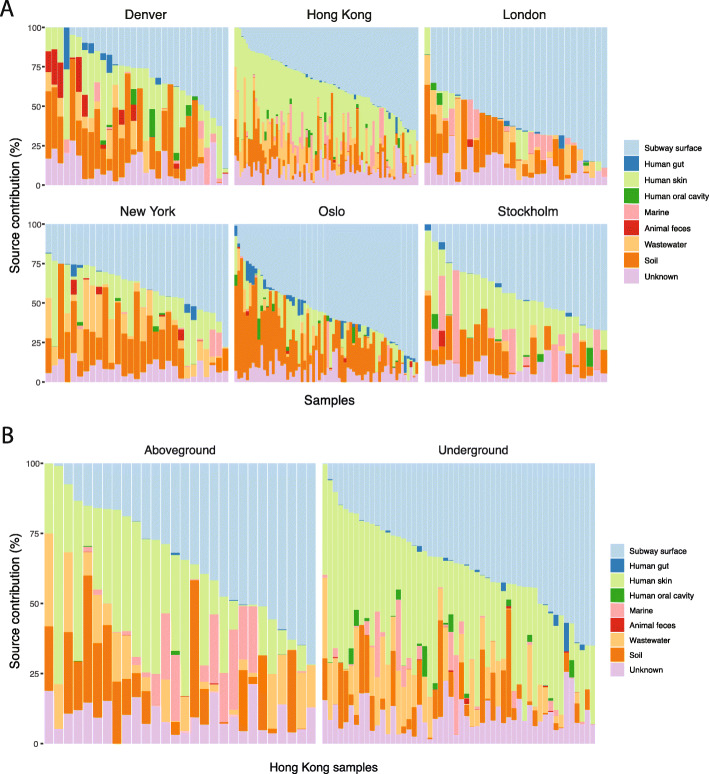
Bayesian sourcetracking estimated public transit surface, human skin, and soil as major AR sources for public transit air resistome. Estimated proportions of resistome sources of different ecotypes in the public transit air microbiomes faceted by city (**a**) and by above- and underground stations within the Hong Kong public transit system (**b**)

We also hypothesized that a major portion of the public transit air resistome would be shared with adjacent public transit surfaces. To this end, we performed a separate SourceTracker analysis, including surface samples collected from the complementary large-scale global public transit microbiome profiling work as putative sources [25]. Geographically specific ShortBRED markers were detected on public transit surfaces with London harbouring the most (466), followed by New York (435), Hong Kong (402), Denver (233), Oslo (223), and Stockholm (160). When public transit surfaces were included, they presented the greater source proportion, surpassing that of human skin (Fig. 5a). Also, compared with aboveground stations, human oral sources overlapped with a greater proportion of the resistome in the air of underground stations in Hong Kong (Mann-Whitney test, *p* = 0.034; Fig. 5b), a pattern not observed for other cities. Overall, the extensive overlap of resistomes between public transit air and those of adjacent public transit surfaces, human skin, soil, and wastewater suggests that these putative sources have major influences on the public transit resistome.

### Assembled contigs detected both AR genes as well as mobile genetic elements

**Fig. 6:**
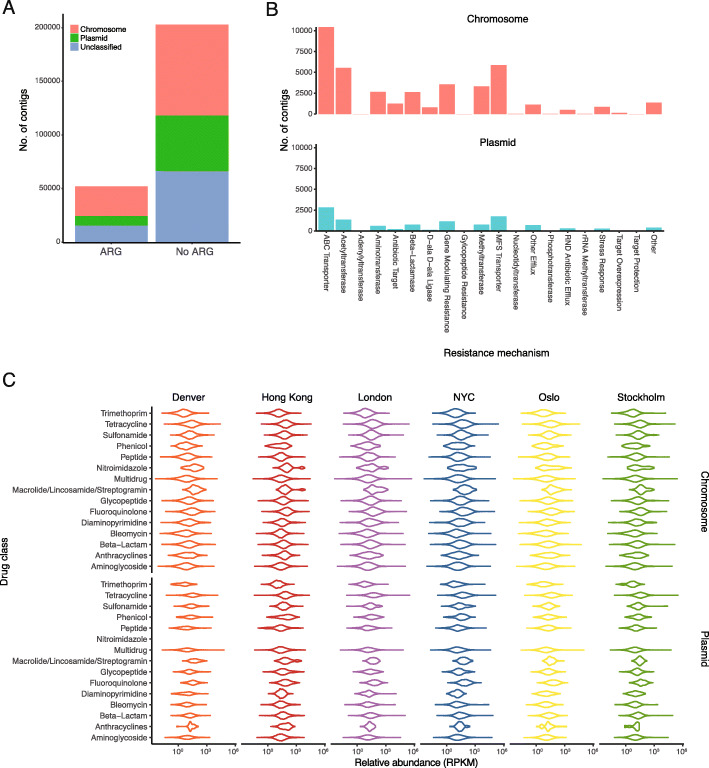
Public transit air resistome contained both chromosome- and plasmid-associated AR genes encoding multiple functional mechanisms of resistance to diverse antimicrobial classes. **a** Detection of AR genes and their genomic context (chromosomal or plasmid-based). **b** Histogram showing the number of contigs containing AR genes encoding genes conferring different mechanisms of resistance, faceted by genetic context in which the AR genes were detected. **c** Abundance data (in RPKM) of genes conferring resistances to different antibiotic classes detected across different cities and genetic contexts

Transmission of AR genes across the public transit air microbiome may be facilitated by mobile genetic elements (MGEs) such as plasmids and integrons [5, 36]. Therefore, identifying the co-localization of AR genes and MGEs will allow a greater understanding on the mobilizable component of the public transit resistome. Co-assembly and dereplication of assembled contigs generated 243,718 non-duplicated contigs with a total of 52,004 (21.3%) containing AR genetic determinants matching the Resfam [37] database. Of the AR gene-containing contigs, 17.1% (8,899/52,004) also contained plasmid determinants (Fig. 6a). Some of these plasmid and/or AR gene-containing contigs also contained integron components such as integrases, *att* sites, and CALIN (cluster of *attC* site lacking integron-integrase) sites (Additional file 8: Table S4). Genes conferring a wide range of resistance mechanisms were detected across chromosomal and plasmid-containing contigs, with genes encoding transporter and acetyltransferase proteins being the most prevalent regardless of genetic context (Fig. 6b). For the antibiotic classes detected, the majority of them were detected in both chromosomal and plasmid-containing contigs, and across all cities (Fig. 6c). Genes conferring resistance to nitroimidazole were not detected on plasmid-containing contigs.

### Metagenome-assembled genome analysis reveals city-unique coding sequences

Following city-based co-assembly, metagenome-assembled genomes (MAGs) were generated. Between Denver, Hong Kong, London, New York, and Oslo, a total of 26 MAGs (genome completeness of ≥ 75% and contamination of ≤ 5%) with taxonomic classification were generated (co-assembled contigs from Stockholm did not generate any MAGs). The MAGs encompassed diverse phyla (Additional file 9: Table S5). MAGs belonging to species commonly associated with humans (e.g. *C. acnes*, *Corynebacterium accolens*, *Micrococcus flavus*, *Dermacoccus nishinomiyaensis*, *Roseomonas mucosa*, and *Kocuria* species), as well as from the environment were detected. We also identified a MAG belonging to *Arsenicicoccus bolidensis*, consistent with a recent work on the Moscow public transit system [38], in which members of this genus were detected.

Reads of each sample were mapped to the 26 MAGs to identify coding sequences (CDSs) in MAGs that were only detected in samples from a particular city (i.e. city-unique CDSs, cuCSDs). A total of 15,523 cuCDSs from the MAGs were identified (Additional file 9: Table S5). The number of cuCDSs ranged from 0.06% (Oslo_bin.107 classified as *Kocuria rosea*) to over 78% (HKG_bin.6 classified as *Actinobacteria* bacterium DSM 45722) of all CDSs in a MAG. The cuCDSs encode broad microbial functions (Additional file 10: Figure S5), with the breadth of functional diversity including resistance to antimicrobials and metals particularly apparent in Hong Kong and Oslo, possibly due to multiple MAGs each containing a high proportion of cuCDSs (e.g. HKG_bin.6 and Oslo_bin.39, Additional file 9: Table S5). MAGs with taxonomic affiliation commonly associated with humans presented a lower percentage of cuCDSs in their genomes (e.g. *C. acnes*, *Micrococcus flavus*, *Lawsonella clevelandensis*, *Kocuria* species, *Dermacoccus nishinomiyaensis*) compared with MAGs with presumptive environmental origins (e.g. *Arsenophonus nasoniae*, *Sandaracinus amylolyticus*, *Azorhizobium doebereinerae*, *Rubrobacter* species). A large number of cuCDSs encode yet unknown functions, suggesting that much of the geographical uniqueness in microbial functional potentials remains to be understood.

### Detection of biosynthetic gene clusters in MAGs from public transit

**Fig. 7.**
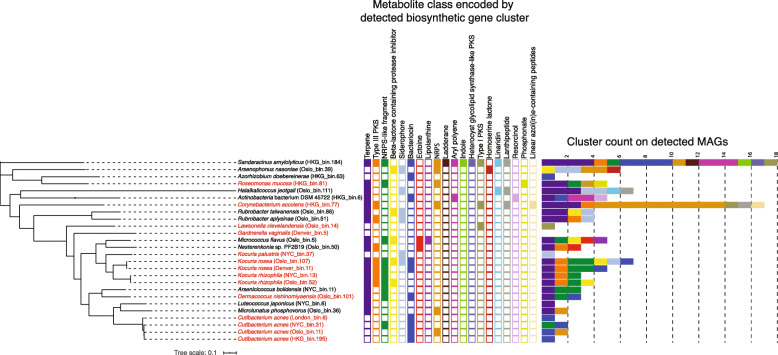
MAGs within the public transit air microbiome contained a diverse collection of gene clusters encoding proteins involved in biosynthesis of secondary metabolites. MAGs with secondary metabolite BGCs. Species known to colonize the human skin, nasal, and urogenital tracts are indicated in red. Types of metabolites synthesized by BGCs in MAGs are indicated by filled tiles. The number of BGCs detected in MAGs, with bars coloured by type of metabolite

Given that genes associated with the synthesis of secondary metabolites were strong indicators for geography-based functional variations (based on FishTaco), characterization of biosynthetic gene clusters (BGCs) in public transit air may inform us of the potential for the expression of secondary metabolites by the public transit microbiome and environments in which the public transit microbiome was sourced. From the 26 MAGs with species taxonomy identified in public transit air, a total of 111 secondary metabolite BGCs were detected, encoding proteins associated with the synthesis of 20 types of metabolites (Fig. 7). The most prevalent BGCs found in MAGs of different taxonomies encode proteins associated with the synthesis of terpenes, bacteriocins, polyketides (polyketide synthases), and those that encode non-ribosomal peptide synthetases (NRPSs) and NRPS-like proteins. MAGs identified as species associated with the human microbiota (Fig. 7, species in red) had a lower average (non-significant difference) of BGCs compared with other MAGs identified. Bacteriocins, which have been shown to be important for competition amongst skin colonizers [39], were detected in MAGs of skin-associated bacteria including *C. acnes*, *K. rosea*, and *Dermacoccus nishinomiyaensis*.

## Discussion

This large-scale and comprehensive characterization of the public transit air microbiome and resistome, employing standardized air sampling as well as centralized sample processing and bioinformatics methodologies, demonstrates a novel approach towards the characterization and mapping of bioaerosols in the BE. We identified factors associated with airborne microbiome composition, microbial functional, and resistance profiles across public transit environments. Our analyses indicated that the public transit air microbiome presents geographical variations that may give rise to differences in functional potentials at both community and sub-species levels. An international study to profile the microorganisms in the air of indoor environments such as the public transit is important, because of not only the potential indoor fomite-mediated and airborne transmission of microorganisms [12, 13] but also the need to understand associations between the public transit environment and its microbiome across geographical locations. Such knowledge will enable scientists to understand how building designs can potentially affect occupants’ health and well-being via changes in the public transit microbiome on a global scale. Our characterization of the public transit air microbiome suggests that pathogens included in the NIAID list were not present or were below the detection limit of the study. However, systematic follow-up investigations with increased sensitivity (e.g. quantitative PCR) can be performed to further examine the abundance of pathogens in the public transit environment. In addition, given that the most abundant species in the study, *C. acnes*, can be considered an opportunistic pathogen [31], careful considerations must be placed in future works involving bioassays and resistance testing to ascertain the risks of exposure to this otherwise human commensal in public transits.

As in other BEs [25, 40–43], different cities appeared to be associated with variations in the composition, the growth profile, functional and AR potentials of the public transit air microbiome. Our resistome sourcetracking observation, where the resistome of public transit air exhibited the greatest similarities with that of the human skin and soil, reinforces the notion that the public transit air microbiome and resistome are predominantly sourced from the outdoors and public transit commuters. The high abundance of skin commensals in public transit air is likely the result of direct skin shedding and particle re-suspension [44]. In addition, we performed a separate sourcetracking analysis including adjacent surface resistomes, as overlapping between the microbiomes of indoor air and nearby surface environments has been documented [45]. We identified that adjacent surfaces, when considered a separate putative resistome source, became the most prominent putative sources of the public transit air resistome. While indoor surface microbiomes in urban environments are known to be predominantly sourced from outdoor air and occupant skin [18, 41], our air sourcetracking results including public transit surface resistome data did not completely remove the contribution from skin and outdoor sources (Fig. 5). Surface microbiomes of BEs may be sourced from environments other than those analysed here [41], and could therefore contribute to surface-unique microbial members in the sourcetracking analysis. In any case, inclusion of surface resistomes alongside other predicted environmental and anthropogenic sources as performed here, in combination with longitudinal sampling efforts [3, 4], could help better understand the flow of AR genes between adjacent environments, commuters, and the public transit air.

Our growth rate estimation results are congruent with previous works predicting bacterial growth in aircraft dust samples in that the inferred growth of most bacteria in indoor air was slow if not negligible [29]. Despite its prevalence and abundance, skin-associated bacteria *C. acnes* and *M. luteus* appeared to be slowly replicating, mirroring the in situ results obtained for these species from their primary habitat [28]. On the other hand, the taxa inferred to be most active in public transit air have environmental origins by taxonomy, likely from adjacent outdoor environments. Activity and cultivability of bacteria have been demonstrated in indoor air [5], but whether these taxa are actively metabolizing in public transit air, or whether they were active in their predominant habitats (e.g. commuter skin, soil, plants) then suspended into public transit air, is not known and cannot be deduced solely from this study. Importantly, the estimated community-level growth rate showed geographical variations, while multiple subspecies of *C. acnes* and *M. luteus* may be active within public transit air of particular cities. These findings further exemplify that geographical specificities in public transit air microbiome extend to not only the community composition but also the predicted species- and strain-level growth (and potentially metabolic) profiles. Future cultivation and metatranscriptomics [46] works will be required to provide a comprehensive assessment of growth profiles and gene expression of taxa in public transit air, so that they can complement the in situ resistome and BGCs results detected in this study.

Public transit air microbiome differences between cities were concomitant with functional differences at community, particularly at species and strain levels. Geographically unique functional pathways contributed by indicator species may reflect differences in functional potentials across the public transits examined. Our random forest analysis between strains of *C. acnes* and *M. luteus* suggests that adaptive genes may be important markers for explaining strain-level microbiome differences in public transit air. In our indicator species and MAGs analyses, the majority of geographical specificities arose due to genes encoding metabolic, replication, transport, and maturation functions. However, future cultivation works are required to discern whether the identified metabolic functions are a reflection of incomplete genomes, partial reconstruction of MAGs present in the communities or a truly biological observation potentially addressing the adaptive capabilities of different public transit air microbiomes. To date, no work has been conducted to assess the effects of sequencing depth and/or a hybrid approach combining short- and long-read sequencing [9], on the extent to which metagenomics reads are assembled into contigs and MAGs for air samples. Given that this is the first study to report MAGs from air metagenomes, optimization of sequencing conditions in the future may allow additional MAGs to be reconstructed. Notwithstanding, the results presented in this study reinforce the notion that geographical variations transcend multiple facets of microbial functions and physiologies.

Most existing works on discerning functional differences in microbiomes do not attempt to identify linkages between taxonomic and functional data [47]. By exploring linkages of these two aspects of the public transit microbiome, we have identified specific taxa estimated to drive the enrichment or attenuation of various functional pathways that defined microbiome differences between the public transits examined. More importantly, by combining our indicator species and FishTaco analyses, both abundant (such as skin-associated bacteria *C. acnes* and *M. luteus*, and environmental taxa *P. stutzeri*) and rare taxa played potential roles in driving functional differences between public transit systems. Furthermore, given that the identified taxa have diverse presumptive sources (collection of human and environmental taxa), a greater understanding of how occupant and adjacent microbiomes feed into the public transit air microbiome will allow us to gain insights not only into the public transit microbial communities from a compositional perspective, but also into how the functional potentials will vary across public transits.

Resistome characterization has been performed previously in public BEs including public transit surfaces [7, 16, 25, 48], and AR genes that were co-associated with MGEs have been characterized in dusts to understand the mobilization potential of AR genes in BEs [5, 49, 50]. We detected both chromosomal-based and MGE-based AR genes across the air of all public transits examined, and genes of all but one drug class (nitroimidazole only found in chromosomes) were detected in both chromosomes and near MGEs. While this is the first resistome characterization of public transit air at a continental scale, results from this study alone will not be able to assess the health risks associated with the transmission of resistant and pathogenic organisms in the public transit [5]. Subsequent works on characterizing the global public transit resistome should also focus on the phenotypic detection of resistance, so as to better inform building and engineering experts of the health implications associated with the dissemination of resistance in public transit air.

There have been recent interests in detecting microbial BGCs that encode proteins synthesizing secondary metabolites (including those with antimicrobial potentials) from different ecosystems [51–54]. To our understanding, this is the first account of the detection of BGCs in BE air. The FishTaco results have identified pathways of secondary metabolite production as among the strongest indicators for geography-based functional variations, suggesting that the abundance variations across public transits in genes responsible for the processing of this class of compounds contributed to geographical specificity. BGCs detected in this study included genes associated with the synthesis of terpenes, bacteriocins, polyketide synthases, and NRPSs, all of which may contain antimicrobial and cell-to-cell interaction potentials [54]. Depending on the presumptive sources of the BGC-containing microbes (e.g. skin, soils, marine sources), the BGCs may play roles in shaping the ecology of the source microbiomes [51–54]. Currently, there is little information regarding the repertoire of BGCs and the expression of secondary metabolites in urban air. Future works should be focused on how the diversity and abundance of BGCs in the public transit air can be influenced by biogeography and other human and building factors.

## Conclusions

In summary, this study presented for the first time an in-depth account of the microbiome and resistome of public transit air across multiple cities. The results highlight the specificities associated with the geography of public transit air microbiomes from community to strain levels. In addition, public transit air is found to be a reservoir of AR genes potentially sourced from commuters and the environment including adjacent public transit surfaces. While informative, further work is warranted in ascertaining the viability of the observed microbial communities, as the detected functional potentials and resistome will not necessarily be manifested phenotypically in this atmospheric environment. Such information could provide greater insights into commuter health risks associated with the transmission of potential pathogens and AR across public transit environments [14, 15]. Future works should also expand upon the current methods and findings to include microbiome and resistome data from additional cities of both developed and developing countries in different climate zones so that comprehensive socioeconomic, clinical, and anthropogenic factors can be included to better explain the observed microbiome differences as recently performed in sewage [55, 56]. Additional public transit factors (e.g. occupancy and ridership, temperature, humidity) should be included in correlative analyses to extend our understanding of how different environmental attributes shape the public transit air microbiome. Longitudinal and seasonal investigations of public transit environments, when integrated with clinical [57] and agricultural [58] microbial community and antibiotics usage data, can broaden our understanding of the roles of time, climate, urbanization rate, ethnicity, population density, and antibiotic use may play shaping the public transit air microbiome and resistome at local, regional, and global scales.

## Methods

### Air sample collection

A total of 259 public transit air samples were collected in Denver (*n* = 30), Hong Kong (*n* = 80), London (*n* = 30), New York (*n* = 29), Oslo (*n* = 64), and Stockholm (*n* = 26) from June to September 2017 (Additional file 11: Table S6). Samples from Denver were collected in the city’s rail and bus system, while samples from the other cities were from subway systems. All samples were collected during weekdays and within working hours (where the occupancy was typical of a working day). Stations were selected based on varying geographical properties/contexts (e.g. proximity to coastline, elevation) and building characteristics (e.g. number of transit connections at a station, indoor/outdoor stations, aboveground/underground stations). A detailed description of how the number of transit connections at a station was defined is provided below. Air samples were collected with SASS 3100 Dry Air Samplers (Research International, Monroe, WA, USA) for 30 min at a flowrate of 300 L/min using electret microfibrous filters. Air samplers were mounted on a tripod with the inlet ~ 1.5 m above floor level and facing downward (45°) to avoid direct deposition of large particles. Air filters were immediately placed into sterile 50-ml conical centrifuge tubes and stored at − 80 °C after each collection day. Field negative control samples (Additional file 11: Table S6) were generated by placing a new filter on the air sampler at the sampling locations and removing it without operating the sampler. Laboratory control samples (Additional file 11: Table S6) were generated by placing a piece of a new filter directly in 10 ml lysis buffer before the filter extraction process.

### Criteria for defining the number of transit connections at a station

The number of transit connections was a cumulative score based on the following criteria: every subway line in and/or out of a station was counted as one transit connection; every train station where an interchange to/from a subway station was logical was counted as a transit connection (i.e. the closest subway station and reasonable walking distance); every bus station (defined as an allocated space for buses and closed for other traffic where several bus lines runs from) where an interchange to/from a subway station was logical, counted as one transit connection; interchange indicated on the subway map between nearby stations, which served different lines, was counted as one connection (regardless of how many lines ran through the nearby station); if a subway line had several different end stations, this was accounted for as it increased the connections to/from the station (e.g. a line with two different end station counted as two lines).

### Sample processing and sequencing

All air samples were shipped on dry ice to a single location (Norwegian Defence Research Establishment FFI, Kjeller, Norway) for processing and DNA isolation according to a previously described protocol [59]. Briefly, filter-collected particulates were extracted into liquid using NucliSENS Lysis Buffer (10 ml, BioMérieux, Marcy-l’Étoile, France), and pelleted by centrifugation (7000×*g*, 30 min). The resulting supernatant and pellet fractions were intermediately separated. The pellet was subjected to additional lysis steps including enzymatic lysis (35 °C, 1 h) with a multi-enzyme cocktail (0.3 mg/ml, MetaPolyzyme, Sigma-Aldrich, St. Louis, MO, USA), followed by mechanical lysis involving bead beating (max intensity, 3 min) in a Mini Bead Beater-8 (BioSpec Products, Bartlesville, OK, USA) using ZR BashingBead Lysis Tubes (0.1/0.5-mm beads, Zymo Research, Irvine, CA, USA) filled with PowerBead Solution and Solution C1 (550 and 60 μl, respectively, Qiagen, Hilden, Germany). Bead tubes were centrifuged (13,000×*g*, 2 min) and inhibitors were removed from the lysate with Solution C2 (250 μl) and C3 (200 μl) according to the Dneasy PowerSoil protocol (Qiagen). The lysed pellet sample was recombined with the original supernatant fraction and DNA isolated according to the manual protocol of the NucliSENS Magnetic Extraction Reagents kit (BioMérieux) with two modifications; magnetic silica suspension volume was increased to 90 μl and incubation time was increased to 20 min. Eight reagent (samples that had gone through the DNA extraction process but not the sampling and filter extraction process), laboratory, and field negative controls and two positive controls (10 μl, ZymoBIOMICS Microbial Community Standard, Zymo Research) were included and processed in parallel with the air samples. The DNA samples were quantified on a Qubit 3.0 Fluorometer (Thermo Fischer Scientific, Waltham, MA, USA) using Qubit dsDNA HS assays (Thermo Fischer Scientific). All DNA samples were shipped on dry ice to the HudsonAlpha Genome Center (Huntsville, AL, USA) for library preparation and paired-end 150-bp shotgun sequencing according to a previously described protocol [16, 25].

### Sequence quality control; taxonomic, functional, and resistome classification; growth rate inference; and identification of contaminating taxa

Adapters were removed from raw sequences using AdapterRemoval (v2.2.2) [60], and quality-filtered using Kneaddata (https://huttenhower.sph.harvard.edu/kneaddata/) with default parameters, using the human genome hg38 and phiX as references to remove human and phiX DNA sequences [17]. MetaPhlAn2 (v.2.6.0) [61] was used to provide species-level taxonomic information to short reads. Based on the MetaPhlAn2 taxonomic classification, the prevalence option and stringent mode of decontam [62] (Oct 2018 release) were used to identify potential contaminating species. Four taxa, *Stenotrophomonas maltophilia*, *Streptomyces albus*, *Afipia broomeae*, and another unidentified species of *Afipia*, were identified as potential contaminants (Additional file 12: Figure S6). With the exception of *S. maltophilia*, the relative abundance of each of the three species was < 0.05% across the experimental samples, and they were deemed negligible to warrant removal in experimental samples. *S. maltophilia*, however, had an average relative abundance of 77.1% in the negative controls, and was abundant in all experimental samples. As a result, co-assembly, binning, and bin refinement were performed for eight negative controls using MetaWRAP [63], resulting in one *Xanthomonadaceae* MAG (> 99.5% completeness, < 0.05% contamination), a classification consistent with *S. maltophilia*. The bin was used as a custom reference to remove reads belonging to the potential contaminant (*S. maltophilia*) from the entire dataset using Kneaddata. Other species-level taxa were also detected in the negative control samples (average relative abundance of 0.06% to 9.5%) (Additional file 12: Figure S6). However, these taxa were not considered contaminants according to decontam and were retained for downstream analyses. Following quality control and human and contamination read removal, a total of 9.5 × 10^10^ bases (average 3.7 × 10^8^ ± 1.2 × 10^8^ bases per sample) or 6.8 × 10^8^ reads (average 2.6 × 10^6^ ± 9.0 × 10^5^ reads per sample) were generated for the entire dataset. MetaPhlAn2 was repeated on the retained clean sequences for taxonomic classification. Taxonomic classification was also performed using Kraken (v2.0.7-beta) [64] and Bracken (v2.5) [65]. The relative abundances of major species *C. acnes* and *M. luteus* were highly congruent between the two classification methods (*C. acnes*: Pearson’s correlation = 0.819, *p* = 4.97 × 10^−64^; *M*. *luteus*: Pearson’s correlation = 0.951, *p* = 1.22 × 10^−133^). The two classification methods also did not affect the interpretation of geographical variations in the overall community composition (see below). Given the recent use of MetaPhlAn2 for another study on urban air microbiomes [49], we decided to adopt MetaPhlAn2 as the classification method for this work. HUMAnN2 (v0.11.1) [66] and ShortBRED (v.0.9.5) [33] were used to profile the functional and resistance potentials of metagenomes, respectively. The Comprehensive Antibiotic Resistance Database (CARD, mid-2017 release) [67] was employed as the reference database to detect and identify AR protein families. The core taxa and resistance protein families were defined as those present in ≥ 75% of the dataset. Species-level growth rates were inferred using GRiD (v1.3) [28], and growth rates were inferred for strains of *C*. *acnes* and *M*. *luteus* (using a SNP-based approach) using SMEG (v1.1.1) [68]. Default settings were applied for both inference tools. GRiD and SMEG values indicate the *ori* to *ter* coverage ratio for a given species or strain, respectively.

### Alpha- and beta-diversity analysis

Clean sequences were rarefied to 316,994 reads per sample using the “seqtk” tool (v.1.3-r106) [69] for taxonomic alpha-diversity analysis. The rarefaction depth corresponded to the sample with the lowest number of reads. Taxonomic richness was calculated as the number of species identified in a sample, and abundance-based Shannon diversity index was calculated using the function “diversity” in R package “vegan” (v2.5.3). The significance of different factors (i.e. city) on the alpha-diversity of public transit air microbiomes was determined using the function “lmer” in R package “lme4” (v.1.1-21). Multiple samples were collected at each subway station (Additional file 11: Table S6) to account for temporal and stochastic variability. Public transit station was thus included as a random effect in the alpha-diversity analysis to account for the effect of pseudoreplication, and for the unequal number of samples among stations. The alpha-diversity post hoc comparisons on city pairings were studied using the “emmeans” function in R package “emmeans” (v.1.4.4). The marginal coefficient of determination (*R*^2^), which computes only the variance of fixed factors explained in the linear mixed model, was calculated using the r.squaredGLMM function in the R package “MuMIn” (v.1.43.15).

Bray-Curtis dissimilarity and Jaccard distance were calculated for the taxonomic composition, functional potentials, and resistance profiles of the public transit air microbiome using the function “vegdist” in the R package “vegan.” Regardless of whether rarefaction was applied, significant differences in public transit air microbiomes among cities were detected (Additional file 13: Figure S7), indicating that the rarefaction depth adopted was sufficient [70]. In addition, the choice of taxonomic classification tool did not change the interpretation of microbiome differences across cities, as geographical factor was still significant in explaining community compositional differences based on Bracken classification (non-rarefied PERMANOVA *F*-value = 25.53, *R*^2^ = 0.28, rarefied PERMANOVA *F*-value = 18.29, *R*^2^ = 0.25, both *p* = 0.005). The significance of community clustering based on MetaPhlAn2, HUMAnN2, and ShortBRED data by public transit networks and environmental factors was tested using the function “adonis.II” in R package “RVAideMemoire” (v0.9-74). In addition, pairwise PERMANOVA comparisons between cities were computed using the function “pairwise.perm.manova” in “RVAideMemoire” for both the community and resistome data. Given the repeated sampling at individual public transit stations, a nested design with restricted permutations was performed to account for pseudoreplication, with factors such as city, Julian day, transit connections, indoor vs. outdoor, aboveground vs. underground, and latitude included as fixed effects in the nested model. To eliminate the imbalance caused by an unequal number of samples between sampling locations, two samples were randomly selected from each location. This resulted in 70 locations from six cities comprising 140 samples that were included in the final statistical analysis.

The indicator value index of taxonomy (i.e. species), function (i.e. KOs), and resistance (i.e. AR protein families) of public transit air microbiomes were determined using the “multipatt” function in R package “indicspecies” (v.1.6.7) [71] with 999 permutational tests. Species, KOs, and AR protein families that were associated with one particular city or groups of cities with sensitivity and specificity both > 90% were defined as strong indicators.

### Strain-level single nucleotide variant

Reads assigned to *C. acnes* and *M. luteus*, the two most abundant species of the public transit microbiome as identified by MetaPhlAn2, were included for strain-level analysis by examining single-nucleotide polymorphisms using StrainPhlAn [72] with the option “relaxed_parameters3”. The strain-level phylogenetic trees were constructed using the R package “ggtree” (v.2.0.1). PanPhlAn (v.1.2.2.3) [73] was used to compare gene content differences between strains of *C. acnes* and *M. luteus* using the very sensitive mode (--min_coverage 1 --left_max 1.70 --right_min 0.30). Jaccard distances were calculated between sample-pairs based on the gene-content output of PanPhlAn, and principal coordinates analysis plots were generated to visualize geography-based strain-level gene repertoire differences between cities. Centroid sequences for each reference species were subjected to Random Forest analysis using the R package “randomForest” (v.4.6-14) [74] to identify geographically specific markers for strain differentiation within the two species. Identified markers were subjected to EggNOG-mapper (v.4.5.1) [75] to convert markers to KO families.

### Functional contributional diversity analysis for geographically specific core metabolic pathways

Within-sample and between-sample diversity were calculated using the Gini-Simpson index and Bray-Curtis dissimilarity metric by the function “diversity” in R package “diverse” (v.0.1.5) and the function “vegdist” in R package “vegan”, respectively. Within a sample, a functional pathway contributed by a single species would result in a low (simple) within-sample contributional diversity, while a function contributed equally by multiple species would result in a high within-sample contributional diversity (complex). If a function is contributed proportionally by the same groups of species across samples, it would result in a low (conserved) between-sample contributional diversity. On the other hand, a function contributed by different groups of species would result in a high (variable) between-sample contributional diversity. For each function, the mean within-sample and between-sample contributional diversity were calculated respectively.

### Identification of taxonomic drivers of functional shifts

To identify taxa driving the observed microbial functional differences between cities, FishTaco (v1.1.3, single-taxa mode) was employed using default settings, with the addition of the “-inf” option to infer the genomic content of taxa detected [32]. For each city, enrichment of functions and identification of taxa driving the functional shifts associated with that city (e.g. sample groups were divided into Denver vs. non-Denver samples to observe taxa driving functional changes associated with Denver) were performed. Species-level taxa with an average relative abundance of ≤ 0.1% according to MetaPhlAn2 and KOs with abundance of ≤ 5 RPKM according to HUMAnN2 were excluded from these analyses.

### Bayesian sourcetracking of microbiomes and resistomes

A total of 182 metagenomic datasets were used as the potential resistome sources of AR genes detected in public transit air (Additional file 14: Table S7). These source samples were chosen to cover diverse ecotypes including gut, skin, and oral cavity from healthy human individuals, animal faeces, soil, wastewater-activated sludge, and marine water/sediments. In addition, 16 datasets from a parallel shotgun metagenomics study of public transit surfaces in each of the same cities [25] (a total of 96 surface datasets from the six cities) were included to represent the public transit surface resistome. With the exception of the marine dataset, 24–30 samples from each source type were selected equally from the three continents (Asia, Europe, and North America). This was performed to account for any potential geography-based heterogeneity in the source resistomes and the resistome data from different geographical locations were combined as one representative global ecotype source. Also, given the dynamic nature of seawater, 31 marine samples were chosen worldwide to potentially reduce regional biases. Raw sequences in .fastq format were retrieved from public databases including NCBI and MG-RAST. Quality filtering, taxonomic, and resistome profiles of the source datasets were processed using the same methods as described above. Representative peptide markers conserved within AR protein families were used for resistome source tracking. The analysis was conducted using the SourceTracker R package [34], with the abundance of markers detected in public transit air samples rarefied to a unique depth of 312 per sample.

### Geographically specific contig assembly and contig dereplication for detection of AR genes and plasmids

Co-assembly of short reads from samples of each city was performed using MegaHIT (v1.1.3) [76]. As primary contigs may be duplicated within contig sets between cities, dereplication of primary contigs was performed as described previously [77]. Briefly, primary contigs ≥ 2000 bp were subjected to CD-HIT-EST (parameter -c 0.99) to generate non-duplicated contigs for secondary contig assembly using Minimus2 (parameters: -D OVERLAP=100 MINID=95). Secondary contigs that were dereplicated (contig sequences with no city name as prefixes in their sequence IDs) and primary contigs that did not assemble in Minimus2 (i.e. geographically specific unique contigs) were combined to form a collection of 243,718 non-duplicated contigs across the dataset. AR genes were detected from these non-duplicated contigs based on the ResFam database as performed previously [78]. Briefly, Prodigal (v2.6.3, default setting) predicted 1,203,035 amino acid sequences from gene-encoding nucleotide sequences within contigs, and amino acid sequences were searched against the ResFam antibiotic resistance gene hidden Markov model database using the *hmmscan* function of HMMER (v3.1b2). Plasmid sequences in contigs were identified using PlasFlow [36] (v1.0), and integron elements were detected using Integron Finder (v.1.5) with default parameters [79]. Contigs containing both AR genes and MGEs were identified as the presence of co-localization. AR gene and plasmid coverage were determined by mapping the short reads against the contigs of each sample to provide coverage information using bbmap.sh (v37.68) (parameters: kfilter=22 subfilter=15 maxindel=80), and pileup.sh was used to convert bbmap coverage data to reads per kilobase per million (RPKM) as described [77].

### Analysis of biosynthetic gene clusters and geographically unique protein clusters from pangenome MAGs

Contigs from geographically specific co-assemblies were subjected to binning and bin optimization steps using MetaWRAP [63]. MAGs were named with the cities from which the contigs originated to construct the MAGs. A total of 26 MAGs with ≥ 75% completeness and ≤ 5% contamination were obtained and assigned taxonomy using PhyloPhlAn2 [80]. On average, approximately 4.71 ± 0.029% of reads from each sample were incorporated into MAGs generated from the same city. A phylogenomic tree of the MAGs was constructed using the “anvi-gen-phylogenomic-tree” command in Anvi’o v6.1 [81, 82] based on the “Bacteria_71” curated hidden Markov Model profile of single-copy genes [83]. Secondary metabolite biosynthesis gene clusters (BGCs) were detected and identified using antiSMASH (v.5.1.1) [84] by the relaxed strictness mode. Graphical representation of the phylogenomic tree and metabolite gene clusters for MAGs were performed using the Interactive Tree of Life (v5) [85]. CDSs from the 26 MAGs detected using Prodigal were subjected to bbmap coverage analysis (bbmap.sh and pileup.sh commands) in order to identify city-unique CDSs (cuCDSs). A cuCDS is defined in this study as a CDS from a MAG constructed from co-assembly of samples from a particular city, in which the coverage of reads is solely from samples of that city and not another city. Identified cuCDSs were subjected to functional annotation using the default settings of eggNOG-mapper online (v2, minimum hit e-value: 0.001, minimum hit bit-score: 60, minimum 20% of query coverage) [86] based on eggNOG v5 clusters and phylogenies [87].

## Supplementary Information

**Additional file 1: Table S1.** Statistical significance of fixed factors selected in determining its roles in microbial diversity and community structure.

**Additional file 2: Figure S1.** Contributional diversity of indicator KOs. **a** Species contribution within Hong Kong public transit to *mtfabH* beta-ketoacyl-[acyl-carrier-protein] synthase III (K11608). **b** Within-sample (defined by Gini-Simpson index) and between-sample (defined by Bray-Curtis dissimilarity) diversity for each within-city core pathway faceted by city. Pathways were colour-coded according to whether the pathway was complex and conserved (red), complex and variable (orange), simple and conserved (green), or simple and variable (blue). **c** Species-level contribution of a simple and conserved pathway (ketogenesis) in Hong Kong, simple and variable (chorismate biosynthesis from 3-dehydroquinate) in New York, complex and variable (preQ0 biosynthesis) in London, and complex and conserved (UDP-N-acetyl-D-glucosamine biosynthesis I) in Stockholm.

**Additional file 3: Table S2.** Contributional diversity of geographically specific core pathways.

**Additional file 4: Figure S2.** Taxonomic drivers for functional shifts associated with public transit systems. The top ten KEGG pathways with the highest functional shift Wilcoxon score (W, diamond signs on figure) for each public transit system: **(a)** Denver, **(b)** Hong Kong, **(c)** London, **(d)** New York, **(e)** Oslo, and **(f)** Stockholm. Vertical lines denote W score of zero, which separate the taxa’s contribution to abundance enrichment (positive score) and depletion (negative score) of a given functional pathway. Each pathway contains two bars of taxonomic information. The top bar for each pathway denote taxa that were enriched in the particular city, and taxa at the bottom bar denote those that were depleted in the city. Additional file 5: Table S3 contains the entire list of differential functions and estimated taxonomic drivers for each function. By default, FishTaco labels *C. acnes* as *P. acnes*. For the purpose of this manuscript, both names are interchangeable.

**Additional file 5: Table S3.** Taxonomic drivers of functional shifts across public transit systems based on KEGG pathways.

**Additional file 6: Figure S3.** Principal coordinates analysis plot of AR protein families based on Bray-Curtis dissimilarity of public transit air microbiomes grouped by city. Figure axes show percentage contribution to overall resistome variations that can be explained by the axes. The normal confidence ellipses indicate the confidence level at 95%.

**Additional file 7: Figure S4.** Bayesian sourcetracking without public transit surfaces as resistome sources. Estimated proportions of resistome sources of different ecotypes in the public transit air microbiomes faceted by city.

**Additional file 8: Table S4.** Detection of integron elements on co-assembled contigs.

**Additional file 9: Table S5.** Species-level taxonomic classification of MAGs, and annotations of city-unique coding sequences.

**Additional file 10: Figure S5.** Proportion of cuCDSs in MAGs grouped by COG functional categories faceted by city. Percentages represent the proportion of cuCDSs belonging to a particular COG category out of all cuCDSs from the same city. Stockholm is not presented in this figure as no MAG was constructed based on samples from that city. Categories are colour-coded based on three general broad functions. Unclassified cuCDSs and those of unknown functions are classified as “Unclassified/Unknown.”

**Additional file 11: Table S6.** Sample metadata for public transit networks.

**Additional file 12: Figure S6.** Taxonomic composition of samples including negative controls. The major 11 species-taxa of the entire dataset are shown with the grey bar at the base of the plot indicating the negative controls.

**Additional file 13: Figure S7.** Community composition variations in public transit air between cities are minimally affected by rarefaction depth. The PCoA plots depict the Bray-Curtis dissimilarity-based community composition variations between cities **(a)** without rarefaction and **(b)** with rarefaction at 316,994 reads per sample. Both sets of results reveal minimal difference in variance explained by the first two dimensions and the significance of the city-based clustering.

**Additional file 14: Table S7**. Source resistome data used in public transit resistomes sourcetracking analysis.

## Data Availability

In-house scripts and input files used to generate figures are publicly available online (https://github.com/mhyleung/mass_transit_air_metagenomics). An associated Core Analysis Pipeline (CAP) from the MetaSUB Consortium can be accessed online (https://github.com/MetaSUB). The raw sequence data has been deposited in the NCBI Sequence Read Archive (SRA) under Bioproject ID# PRJNA561080 and is also available online (https://pngb.io/metasub-air-2021).
