# Supplementary figures and images for: Characterization of the public transit air microbiome and resistome reveals geographical specificity
Source: Microbiome. 2021 May 26;9:112. doi: 10.1186/s40168-021-01044-7 (PMC8157753; doi:10.1186/s40168-021-01044-7)

a

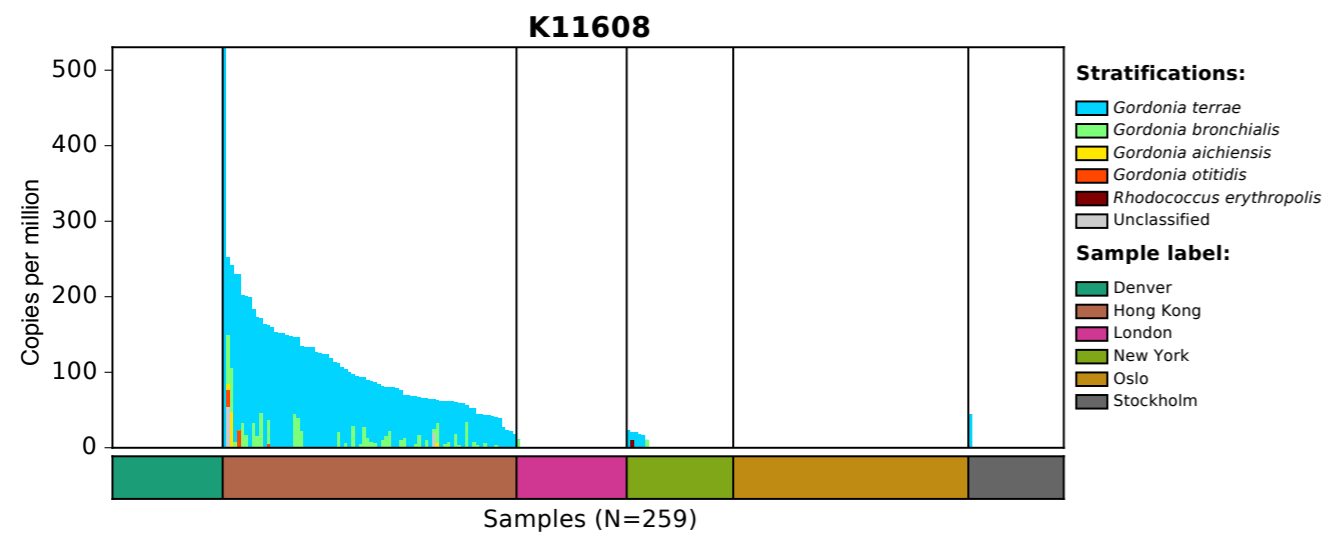

b

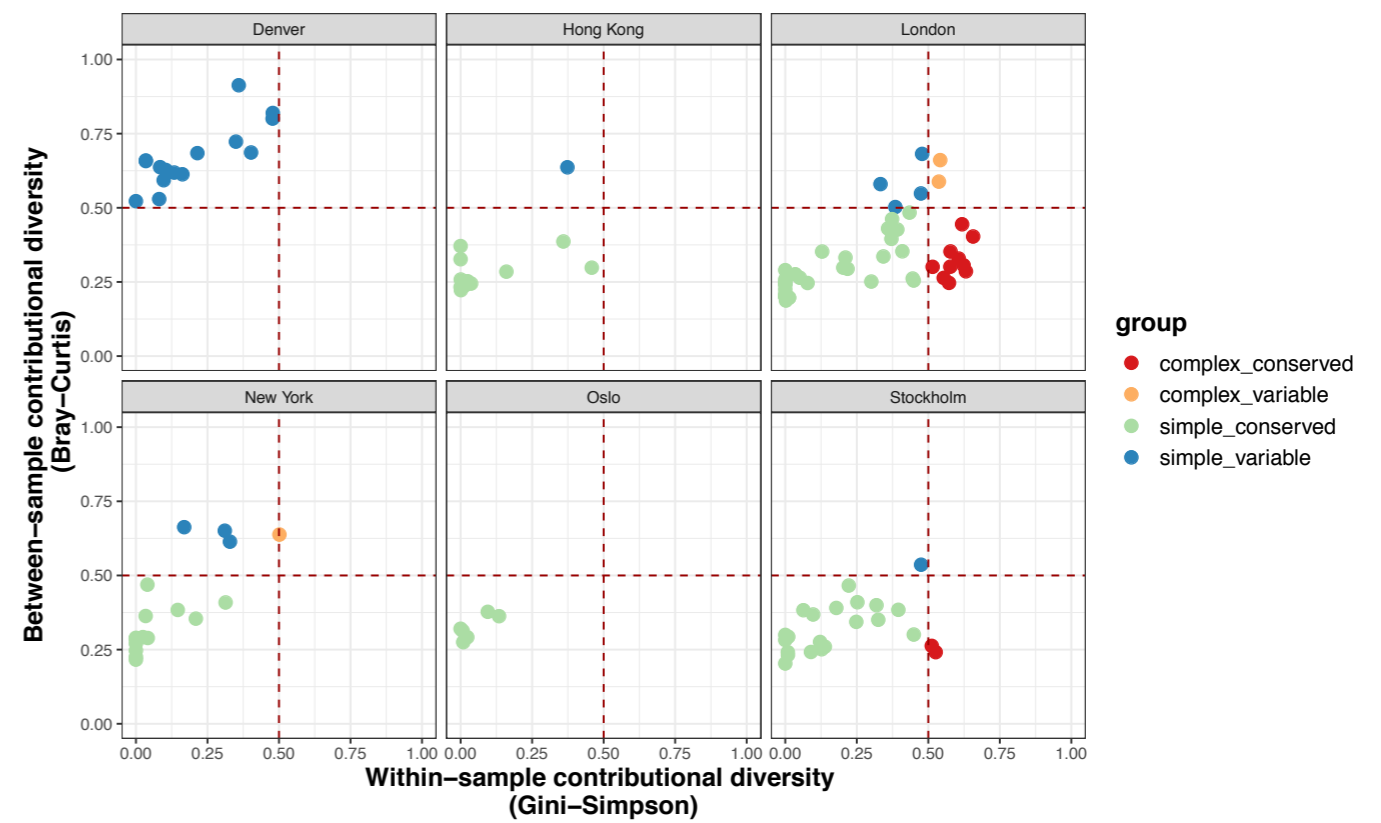

c

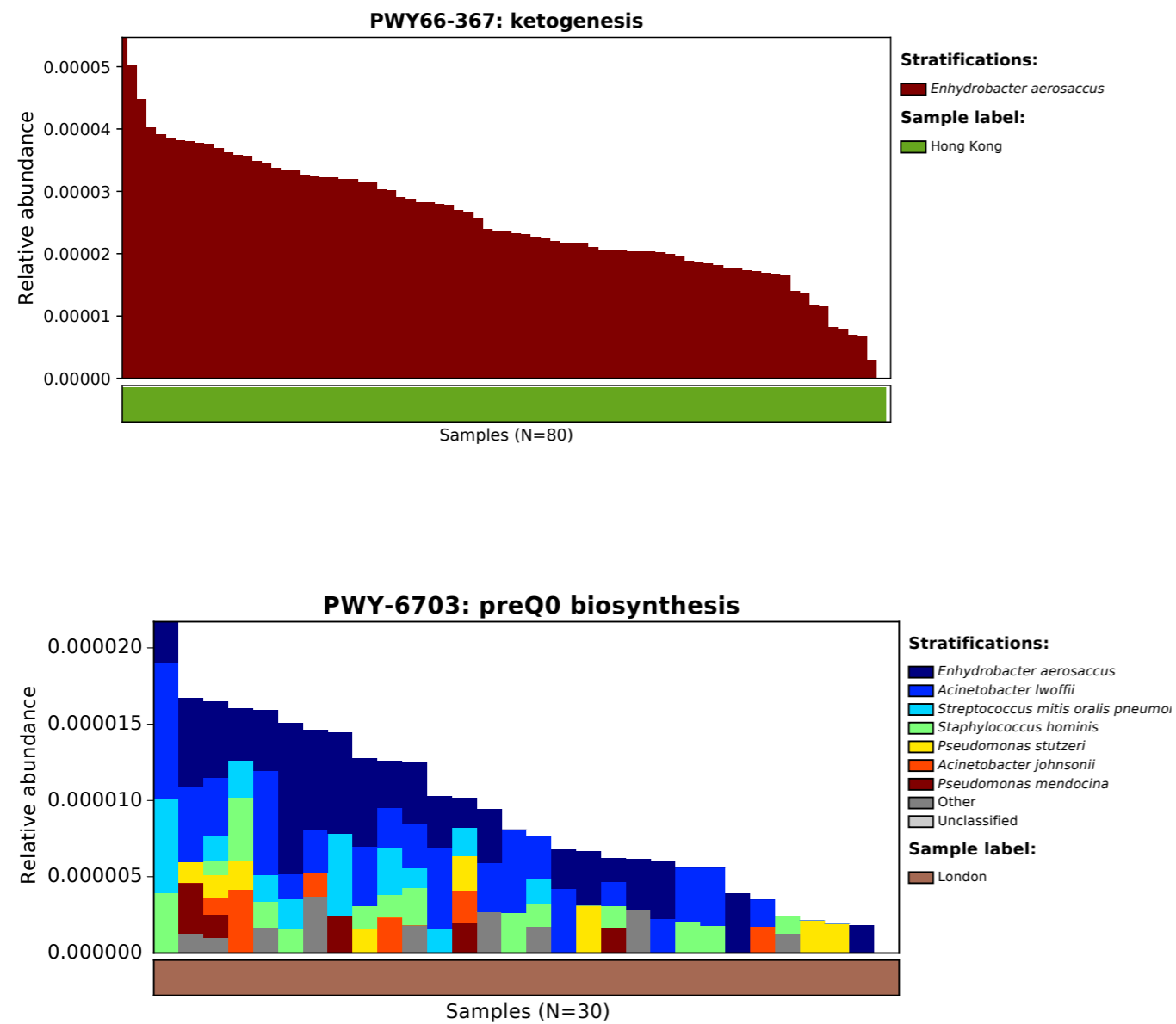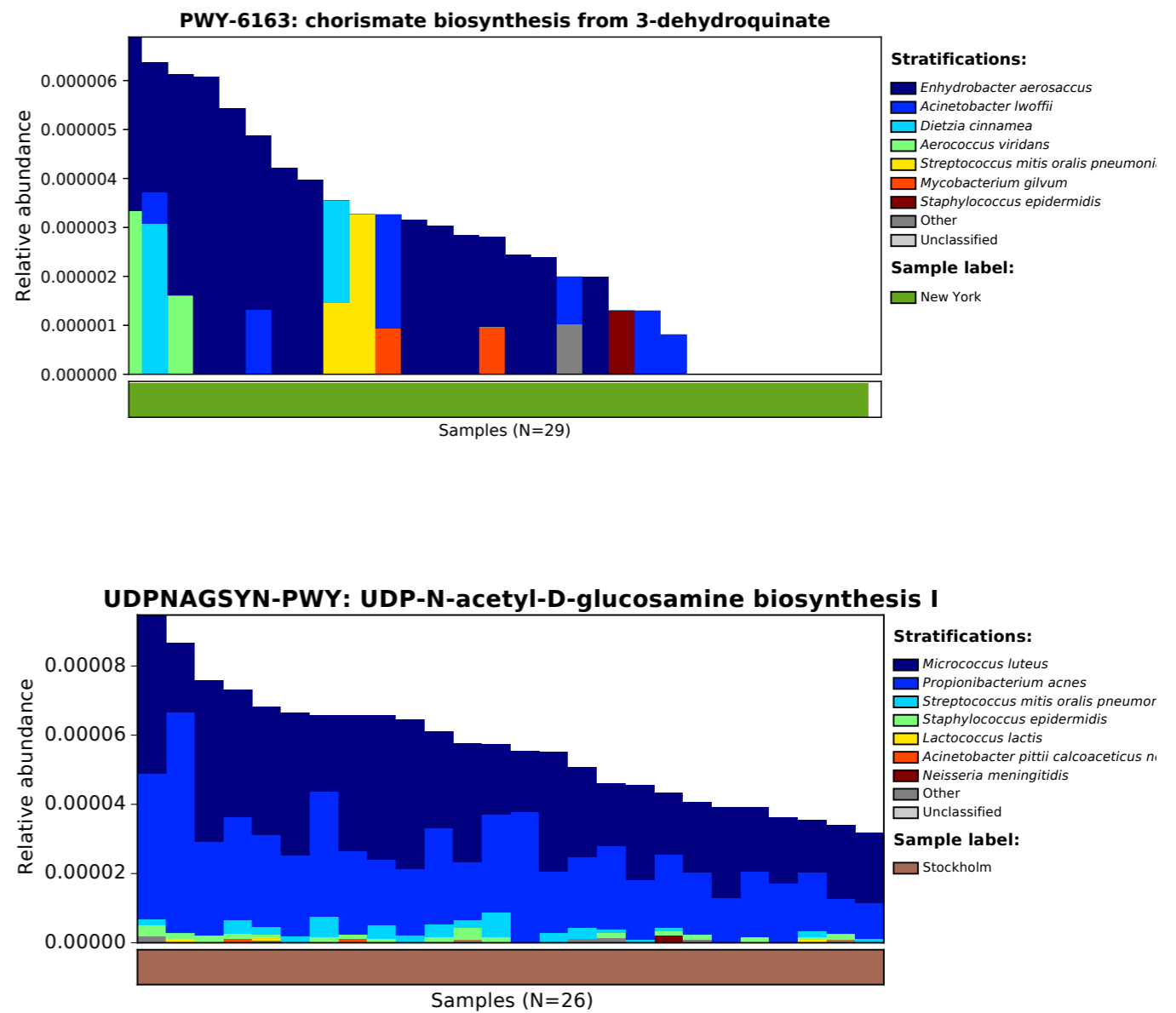

Supplement: Supplementary file 2 — Additional file 2: Figure S1. Contributional diversity of indicator KOs. a Species contribution within Hong Kong public transit to mtfabH beta-ketoacyl-[acyl-carrier-protein] synthase III (K11608). b Within-sample (defined by Gini-Simpson index) and between-sample (defined by Bray-Curtis dissimilarity) diversity for each within-city core pathway faceted by city. Pathways were colour-coded according to whether the pathway was complex and conserved (red), complex and variable (orange), simple and conserved (green), or simple and variable (blue). c Species-level contribution of a simple and conserved pathway (ketogenesis) in Hong Kong, simple and variable (chorismate biosynthesis from 3-dehydroquinate) in New York, complex and variable (preQ0 biosynthesis) in London, and complex and conserved (UDP-N-acetyl-D-glucosamine biosynthesis I) in Stockholm. [file 40168_2021_1044_MOESM2_ESM.pdf]

a

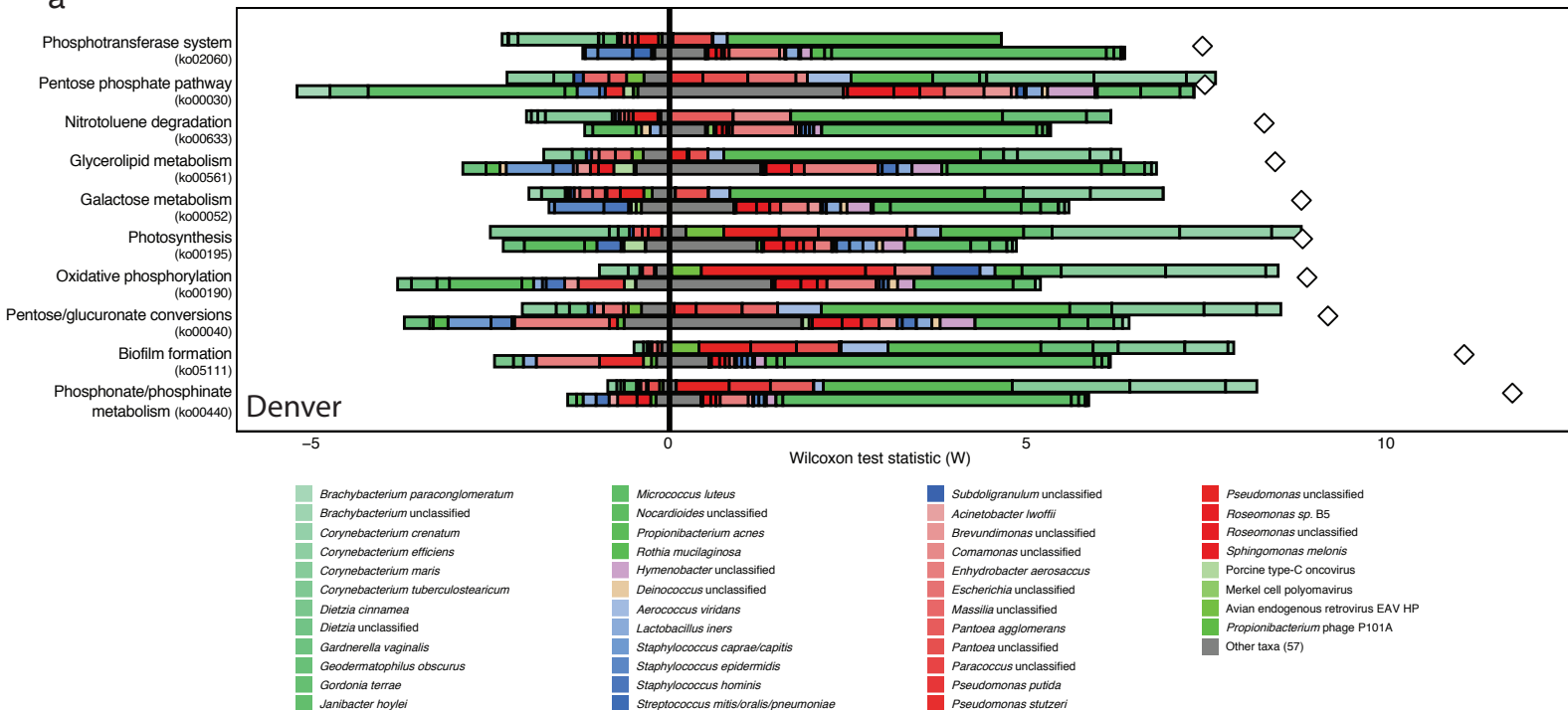

b

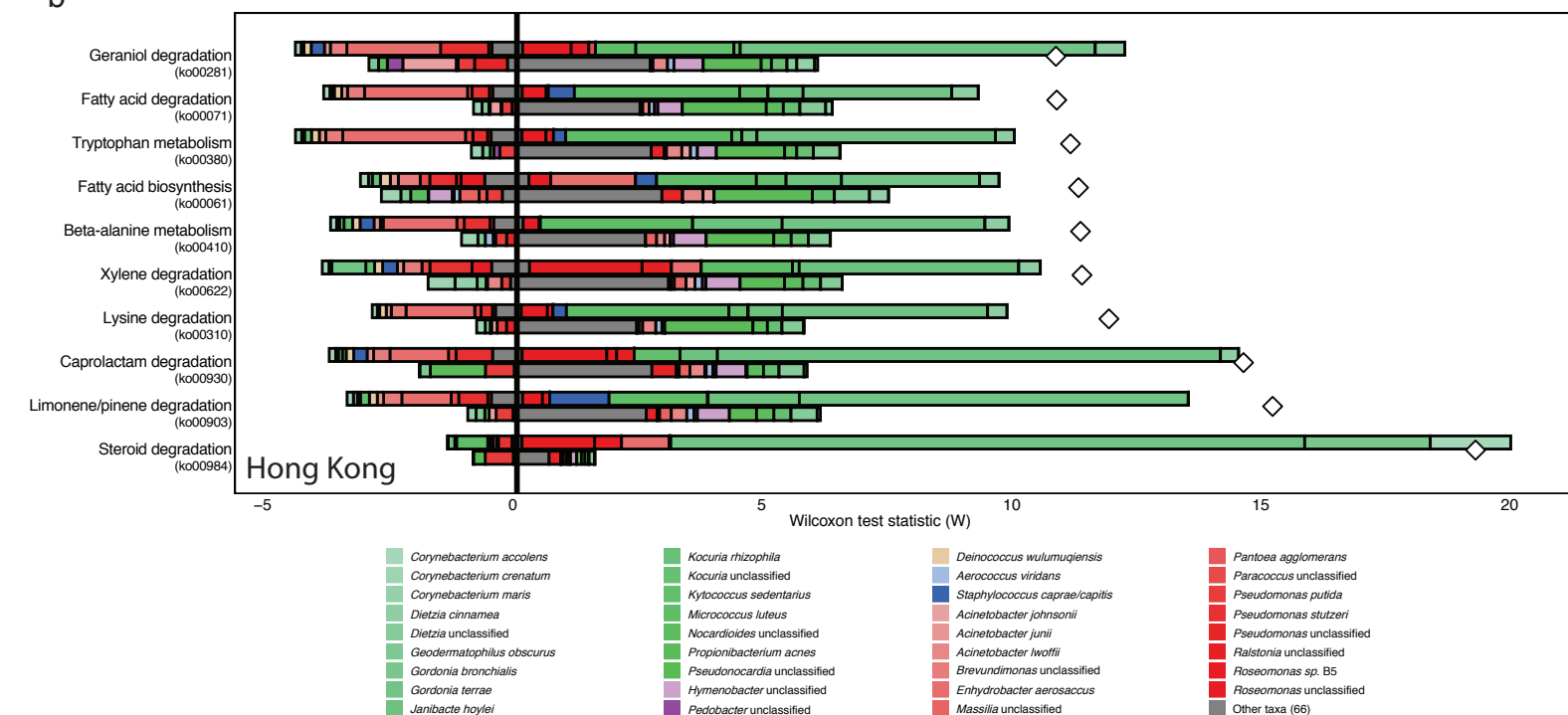

c

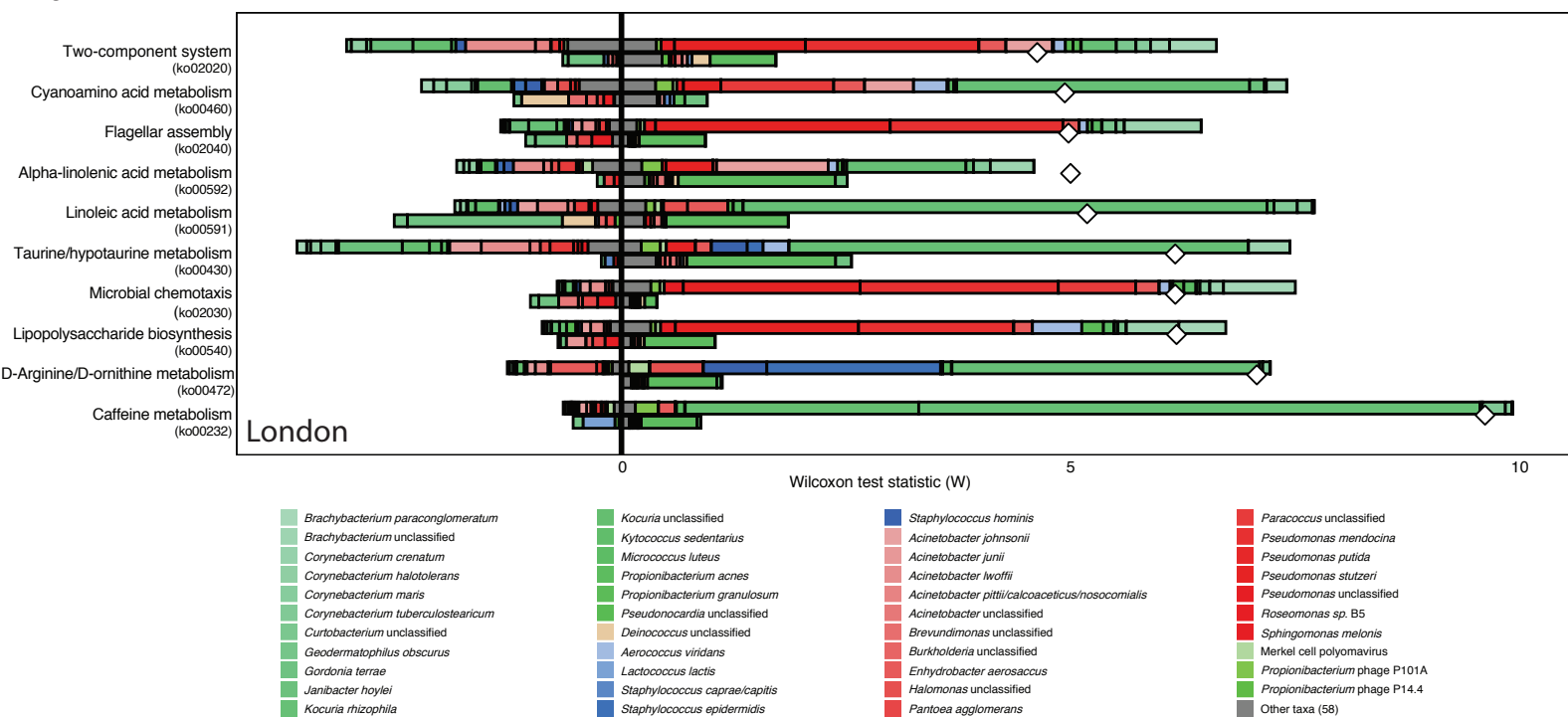

d

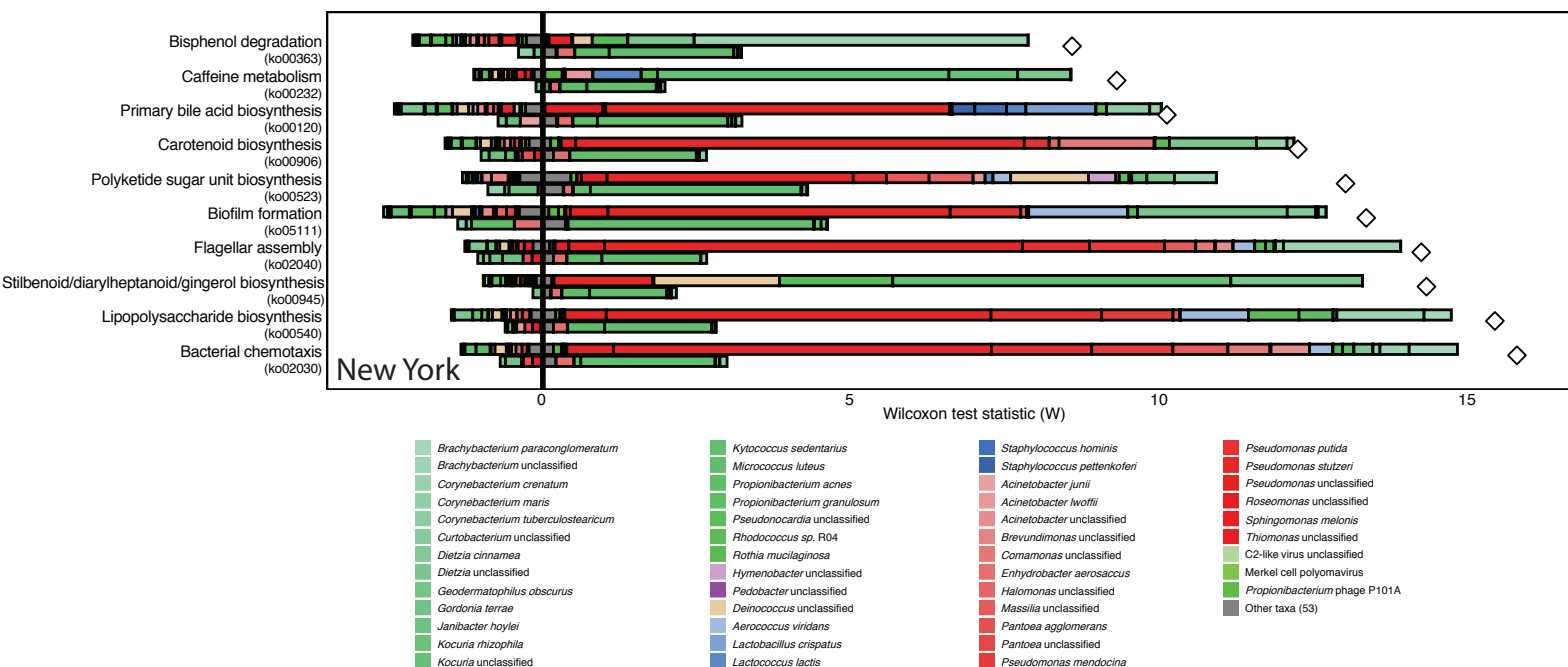

e

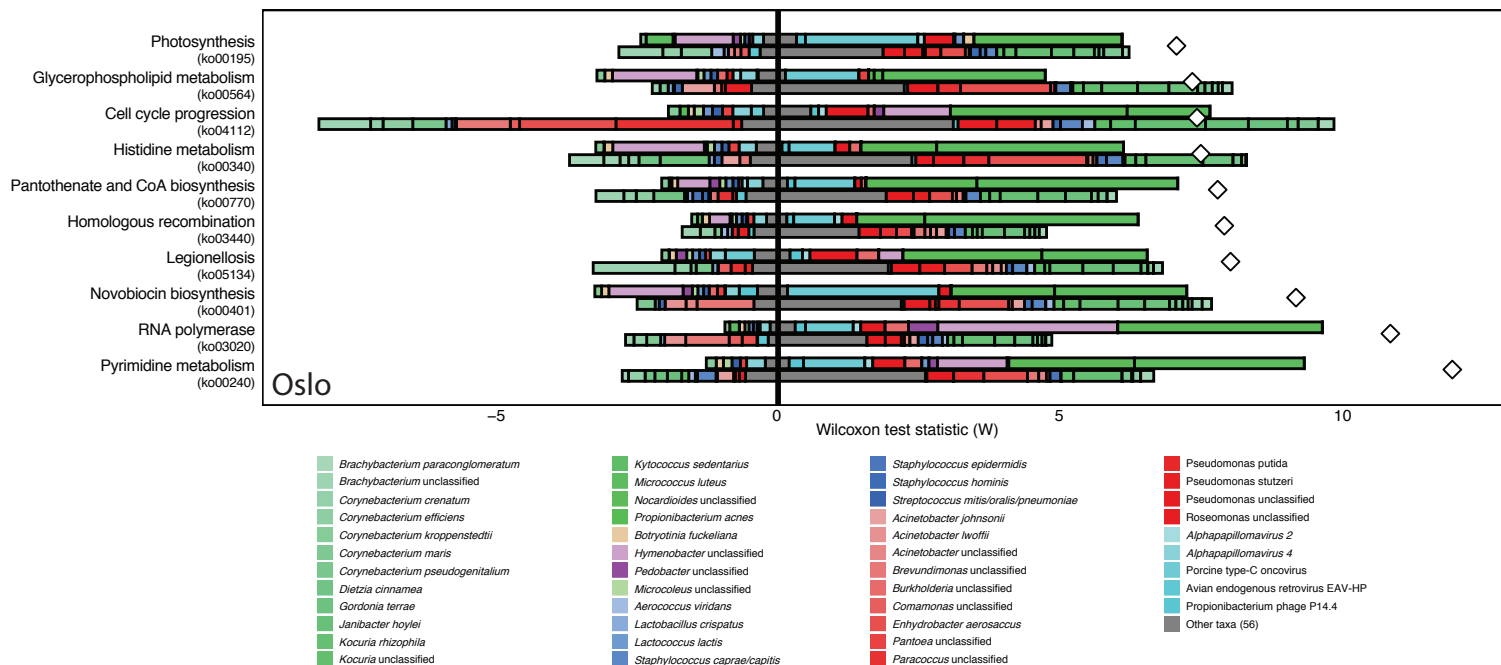

f

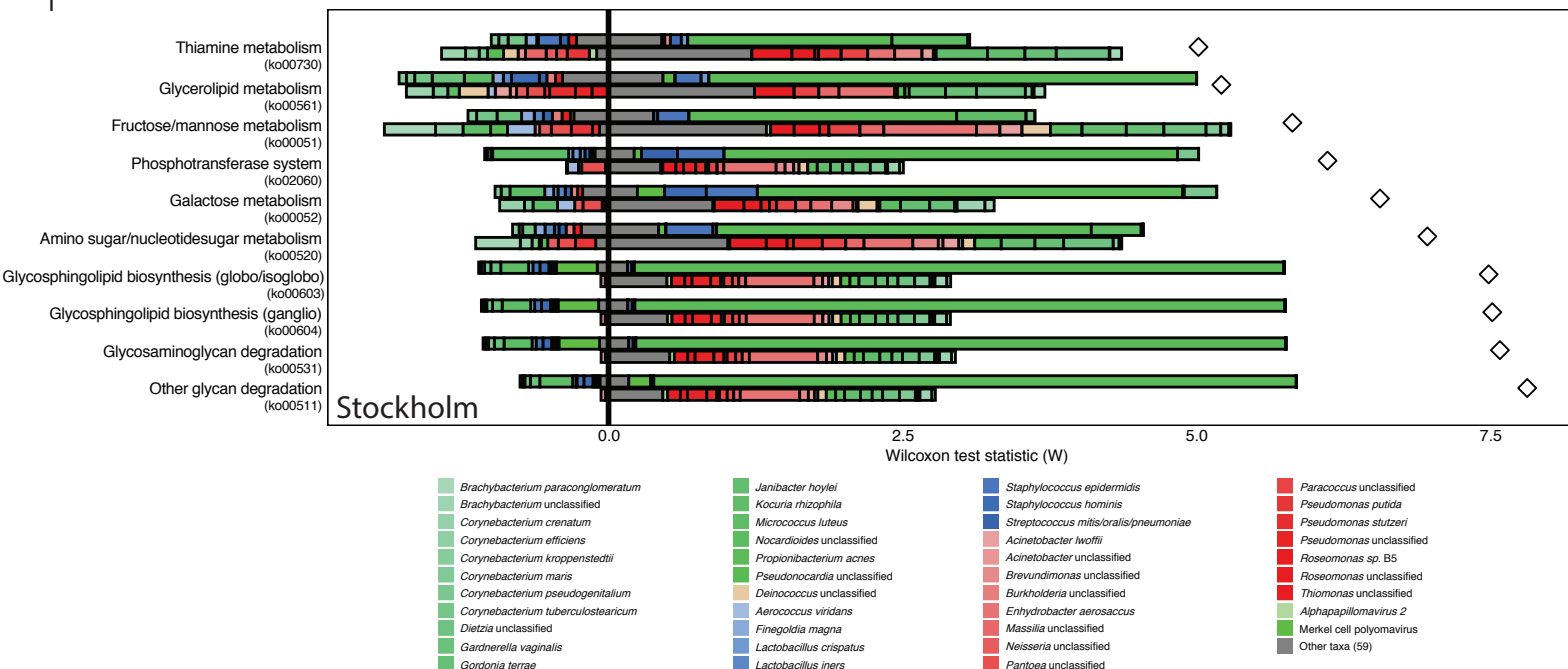

Supplement: Supplementary file 4 — Additional file 4: Figure S2. Taxonomic drivers for functional shifts associated with public transit systems. The top ten KEGG pathways with the highest functional shift Wilcoxon score (W, diamond signs on figure) for each public transit system: (a) Denver, (b) Hong Kong, (c) London, (d) New York, (e) Oslo, and (f) Stockholm. Vertical lines denote W score of zero, which separate the taxa’s contribution to abundance enrichment (positive score) and depletion (negative score) of a given functional pathway. Each pathway contains two bars of taxonomic information. The top bar for each pathway denote taxa that were enriched in the particular city, and taxa at the bottom bar denote those that were depleted in the city. Additional file 5: Table S3 contains the entire list of differential functions and estimated taxonomic drivers for each function. By default, FishTaco labels C. acnes as P. acnes. For the purpose of this manuscript, both names are interchangeable. [file 40168_2021_1044_MOESM4_ESM.pdf]

PCoA2 (variance explained: 9.9%)

0.2  
0.0  
-0.2  
-0.4

PCoA1 (variance explained: 22%)

Additional File 6: Figure S3

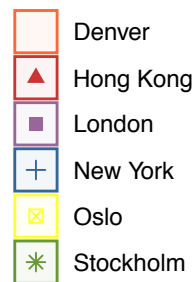

Supplement: Supplementary file 6 — Additional file 6: Figure S3. Principal coordinates analysis plot of AR protein families based on Bray-Curtis dissimilarity of public transit air microbiomes grouped by city. Figure axes show percentage contribution to overall resistome variations that can be explained by the axes. The normal confidence ellipses indicate the confidence level at 95%. [file 40168_2021_1044_MOESM6_ESM.pdf]

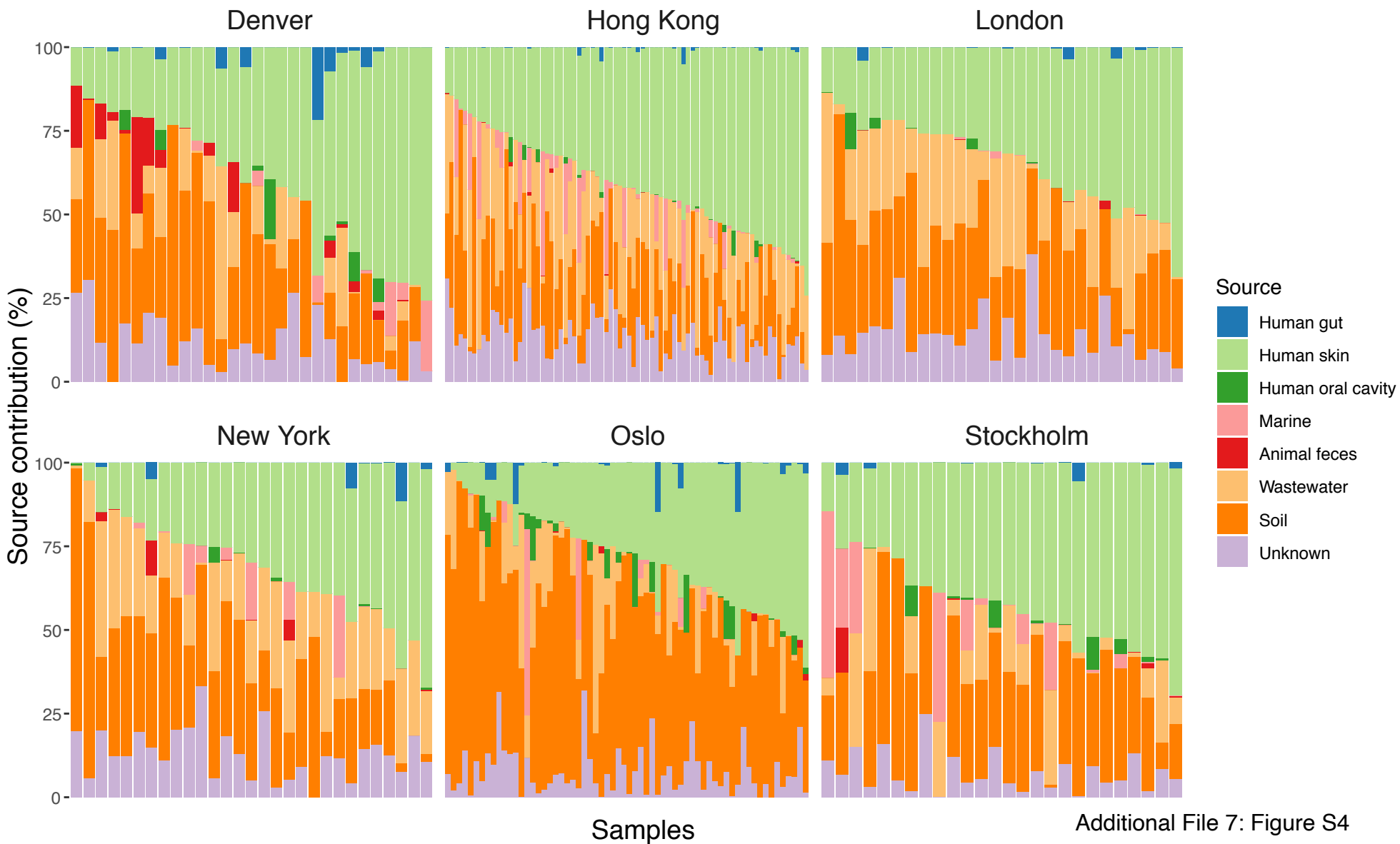

Supplement: Supplementary file 7 — Additional file 7: Figure S4. Bayesian sourcetracking without public transit surfaces as resistome sources. Estimated proportions of resistome sources of different ecotypes in the public transit air microbiomes faceted by city. [file 40168_2021_1044_MOESM7_ESM.pdf]

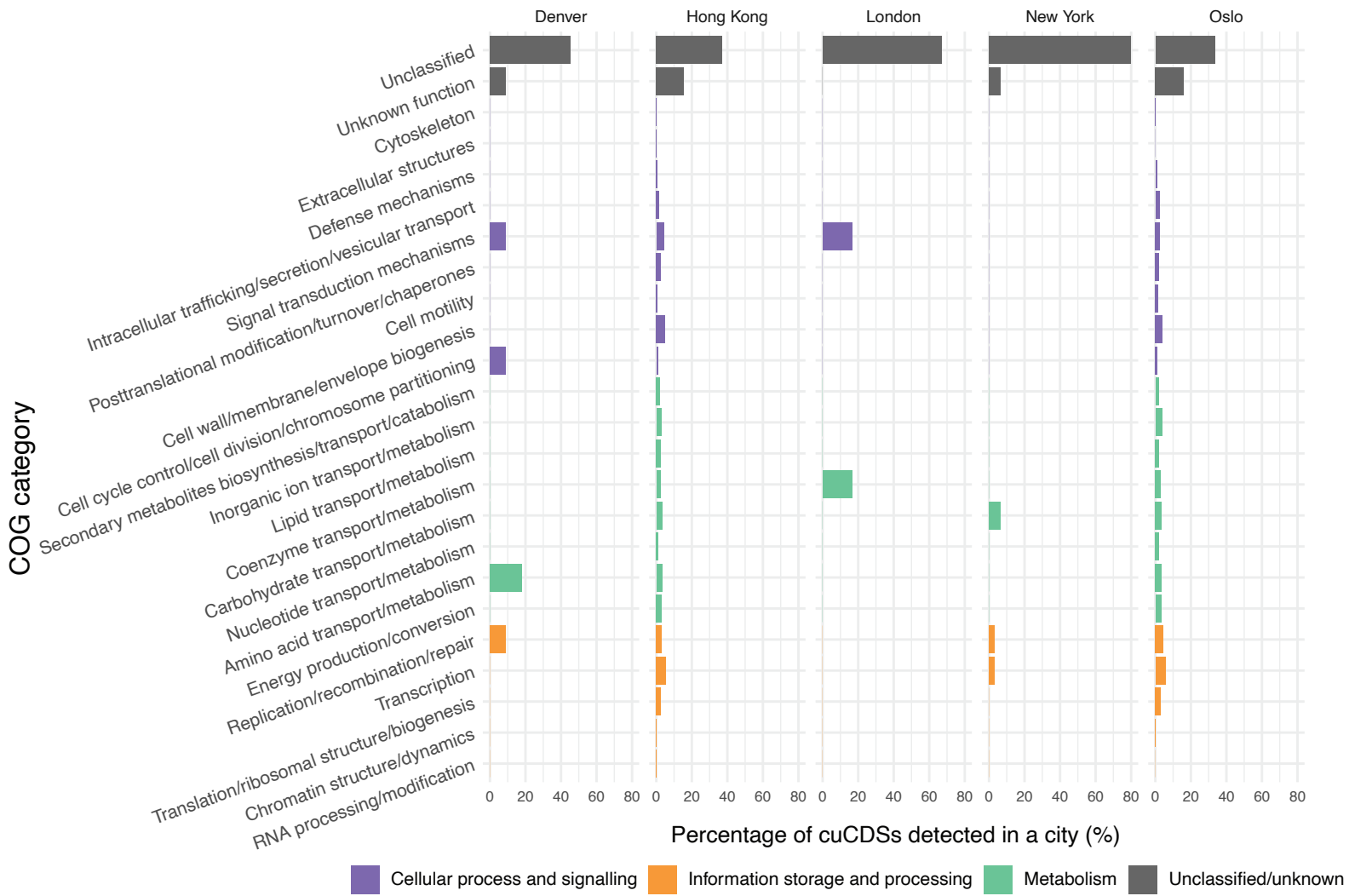

Additional File 10: Figure S5

Supplement: Supplementary file 10 — Additional file 10: Figure S5. Proportion of cuCDSs in MAGs grouped by COG functional categories faceted by city. Percentages represent the proportion of cuCDSs belonging to a particular COG category out of all cuCDSs from the same city. Stockholm is not presented in this figure as no MAG was constructed based on samples from that city. Categories are colour-coded based on three general broad functions. Unclassified cuCDSs and those of unknown functions are classified as “Unclassified/Unknown.” [file 40168_2021_1044_MOESM10_ESM.pdf]

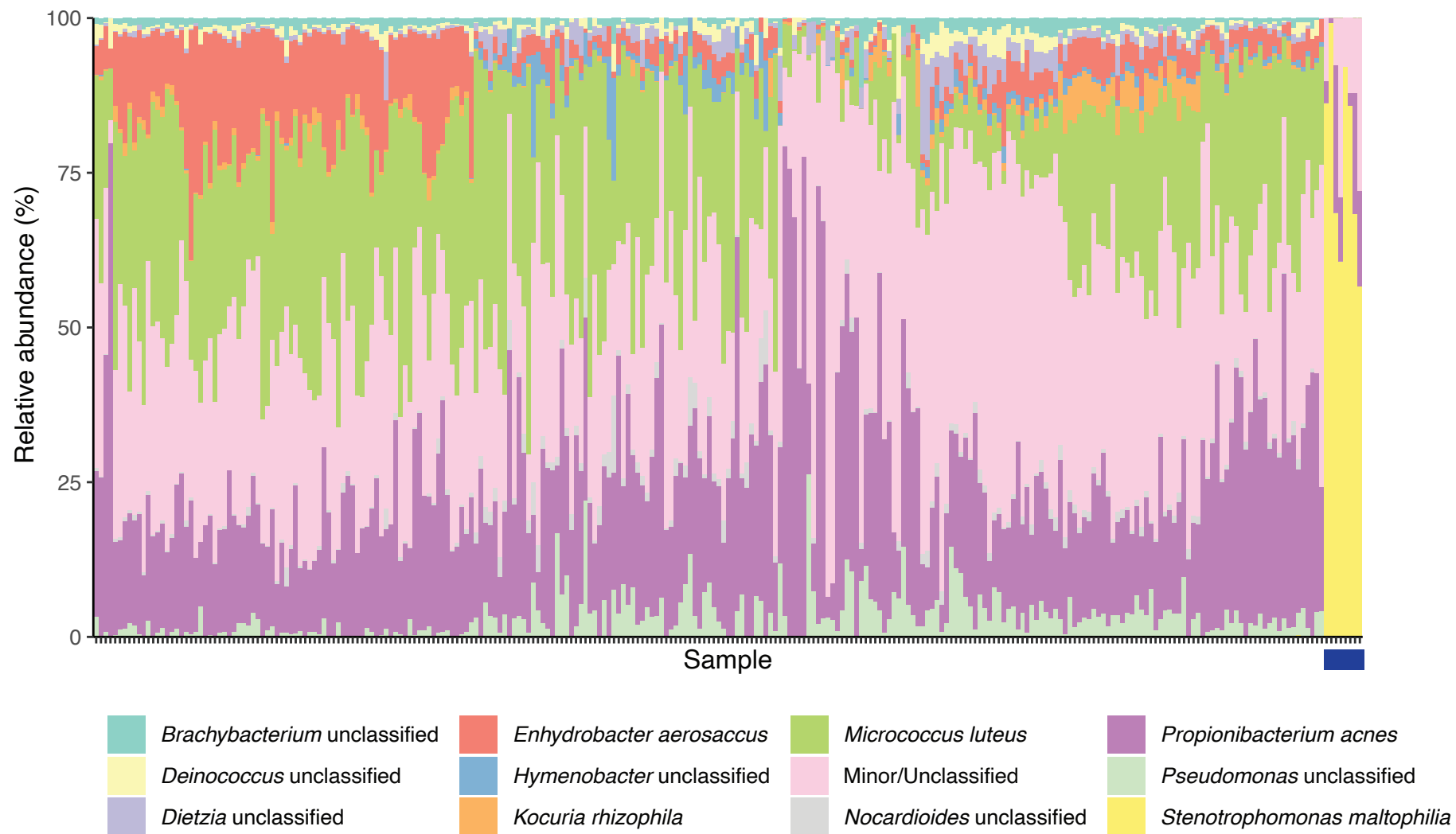

Supplement: Supplementary file 12 — Additional file 12: Figure S6. Taxonomic composition of samples including negative controls. The major 11 species-taxa of the entire dataset are shown with the grey bar at the base of the plot indicating the negative controls. [file 40168_2021_1044_MOESM12_ESM.pdf]

a

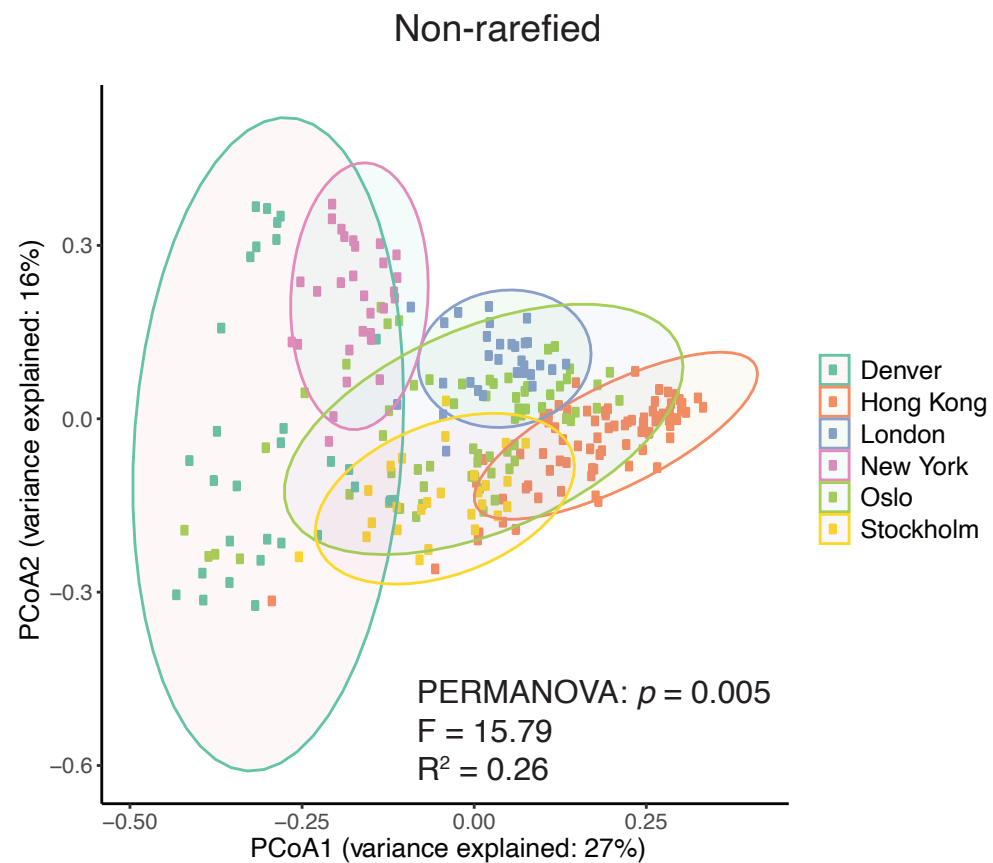

b

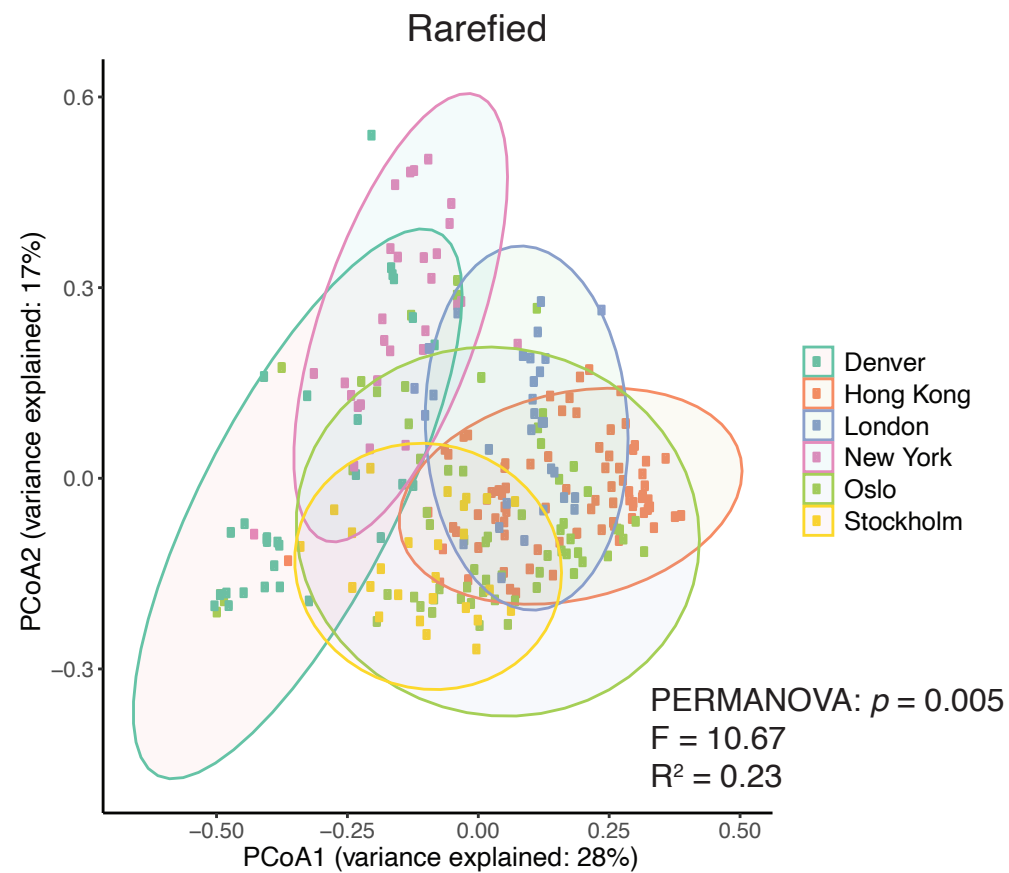

Supplement: Supplementary file 13 — Additional file 13: Figure S7. Community composition variations in public transit air between cities are minimally affected by rarefaction depth. The PCoA plots depict the Bray-Curtis dissimilarity-based community composition variations between cities (a) without rarefaction and (b) with rarefaction at 316,994 reads per sample. Both sets of results reveal minimal difference in variance explained by the first two dimensions and the significance of the city-based clustering. [file 40168_2021_1044_MOESM13_ESM.pdf]
